# The Back Belief Questionnaire is efficient to assess false beliefs and related fear in low back pain populations: A transcultural adaptation and validation study

**DOI:** 10.1371/journal.pone.0186753

**Published:** 2017-12-06

**Authors:** Arnaud Dupeyron, Charlotte Lanhers, Sophie Bastide, Sandrine Alonso, Matthias Toulotte, Claire Jourdan, Emmanuel Coudeyre

**Affiliations:** 1 Service de Médecine Physique et de Réadaptation, Centre Hospitalier Universitaire de Nîmes, Nîmes, France; 2 EuroMov, Université de Montpellier, Montpellier, France; 3 Département de Médecine Physique et de Réadaptation, Centre Hospitalier Universitaire, Clermont-Ferrand, France; 4 Département de Biostatistique, Epidémiologie, Santé Publique et Informatique Médicale (BESPIM), Centre Hospitalier Universitaire de Nîmes, Nîmes, France; 5 Département de Médecine Physique et de Réadaptation, Centre Hospitalier Universitaire Lapeyronie, Montpellier, France; 6 INRA, Unité de Nutrition Humaine (UMR 1019), CRNH Auvergne, Clermont-Ferrand, France; University of Glasgow, UNITED KINGDOM

## Abstract

**Background:**

According to the fear avoidance model, beliefs and thoughts can modify the outcome of patient with low back pain. The Back Belief Questionnaire (BBQ)–a 14 items scale–assesses these consequences of low back pain.

**Objective:**

To test the psychometric properties of the French version of the BBQ.

**Methods:**

The BBQ was translated using the forward–backward translation process. Throughout three repeated evaluation time points (D1, D7 and D30), various aspects of validity were analysed: acceptability, quality of items, unidimentionality, internal consistency, temporal stability (between D1 and D7), responsiveness (between D7 and D30), and construct validity comparing it to other validated scales.

**Results:**

One hundred and thirty-one patients were enrolled and 128 were analyzed. The acceptability and the quality of the items were excellent. The scale was unidimensional and reliable (internal consistency: Cronbach’s α = 0.8). The responsiveness was moderate but in line with other scores. The BBQ was, as expected, convergent with day-to-day activities and fear avoidance (FABQ and Tampa), disability (Quebec and Dallas scores), or anxiety and depression (HAD); and not correlated with pain. Best correlations were found with Tampa and FABQ. The temporal stability (test-retest reliability) was poor. However, similar changes were observed in near conceptual score (FABQ), which confirmed that clinical status may have not been stable and suggesting sensitivity to early changes for BBQ.

**Conclusions:**

The BBQ showed good psychometric properties to assess false beliefs and related fear in French or English LBP populations and can be used either for evaluation in international trials or as a part of self-care training.

## Introduction

According to the bio-psychosocial model, the course of chronic low back pain (CLBP) is widely influenced by emotional, cognitive and behavioral factors [1]. It is well known that cortical processes are involved in the integration of multidimensional aspects of pain. This highlights the shift from mechanical to functional response and leads to cognitive and behavioural adaptation when pain persists. Therefore, patients may adopt individual strategies depending on expectations, fears, and beliefs [2]. There is increasing evidence that beliefs as well as thoughts are widely altered in LBP patients [3] but also in physicians [4]. It is also clear that beliefs can change the way that patients struggle for recovery and autonomy [5].

Symonds *et al*. have developed a specific self-reported questionnaire–the Back Beliefs Questionnaire (BBQ)–designed to explore beliefs and thoughts related to low back pain [6]. Unlike the Fear Avoidance Beliefs Questionnaire (FABQ) score, which explores beliefs related to consequences of LBP on physical and work activities avoidance, the objective of the BBQ is to determine the presence of various inevitable consequences of LBP in patient’s future among 14 determinants. The first validation of the English version only comprised reliability and consistency in individuals and workers and showed that BBQ was able to distinguish workers with false beliefs associated with longer work absenteeism [6]. The BBQ seems to be used in practice, usually in self-care and multidisciplinary programs probably as far as an evaluation (seeking false beliefs) or educational tool (treating false beliefs) [7]. However, this questionnaire has not been tested in non-worker LBP patients and likely needed more extensive validation process.

The aim of this work was to provide a French transcultural validated version of the BBQ. The study was divided into two steps: i) translation and cultural adaption of the BBQ and ii) validation of the French version in terms of acceptability, quality of the items, unidimensionality, internal consistency, temporal stability, responsiveness, and construct validity.

## Method

### The BBQ

Pain-related fear is known to affect daily activities and the development of disability as patients elaborate unsuitable representations of danger, either painful, crippling or destructive and the usefulness of the majority of treatments. Therefore, the items have been designed in order to explore the degree of agreement of patients about developing various inevitable status related to LBP in the future. The questionnaire consists in a 14-item beliefs score. Nine items are used for the score (Q1, Q2, Q3, Q6, Q8, Q10, Q12, Q13 and Q14) and five are used as distractors (Q4, Q5, Q7, Q9 and Q11). The level of each belief ranks from total disagreement to total agreement on a Likert 5-level scale. The score obtained for each item is reversed (e.g. 5 means 1 and 2 means 4) and nine items included in the total score are added. For each item, the higher score means the worst future perceived (either illness perception or treatment effectiveness). For the entire BBQ, the higher the patient scores, the less he displays fear and false beliefs (as scores are reversed).

### Translation

The BBQ was transculturally translated using the forward / backward procedure [8,9] Three French native bilingual physicians (AD, EC, AG) independently translated the questionnaire. They were asked to provide a global rather than word for word translation [10]. They reviewed each translation together for cultural adaptations and obtaining a consensus version. A backward translation into English was then proposed by a native English translator (CS) to check the meaning of each item.

### Population

Patients were eligible if i) they were consulting for back pain condition lasting more than 3 months, ii) previous treatments (medications and/or physiotherapy) had been ineffective and iii) they had no previous history of surgery or multidisciplinary rehabilitation program or dedicated educational intervention. All patients signed an informed consent. The study was approved by the regional ethics committee (Comité de Protection des Personnes Sud Méditerranée III, 2011.06.05), recorded by French authorities (RCB ID n° 2011-A00270-41 delivered by AFSSAPS), and declared on Clinical trials (NCT01389999).

### Study design

To validate the French version of the BBQ, this questionnaire was included in a multidimensional evaluation of CLBP integrated in rehabilitation programs provided by two tertiary care University Hospitals. The questionnaires were filled out on the day of enrolment (D1), on the first day of the rehabilitation program (D7) and one month after the end of the rehabilitation program (D30). No treatment or intervention was scheduled between D1 and D7. The D30 session aimed at controlling the beliefs' changes if any.

### Objective of the study: Validation of the translated BBQ

The validation process consisted in the assessment of the i) acceptability of each item and of the global questionnaire; ii) quality (absence of saturation, ceiling or floor effect, and redundancy) of each item and of the global questionnaire; iii) unidimensionality of scale; iv) internal consistency; v) temporal stability using a test-retest reliability method between D1 and D7 in strictly the same conditions; vi) responsiveness between D7 and D30; and vii) construct validity using correlations with other validated questionnaires exploring different dimensions to assess convergence and divergence.

The other validated questionnaires used were: the FABQ for fear and avoidance [11,12], the Quebec scale for disability [13], HADs for anxiety and depression [14], the Tampa for kinesiophobia [15], Visual analogue scale (VAS) for the pain, and the Dallas pain questionnaire for day-to-day activities [16]. At D1 only the BBQ, Tampa, Quebec and FABQ were recorded in order to control for changes in close concepts (fear and kinesiophobia), all questionnaires were administered at D7 and D30.

### Statistical analysis

Acceptability was assessed by the number and the proportion of the overall and for each item absence of responses (coted “no”). Acceptable items have to provide a proportion of “no” responses lower than 5%, if the proportion is higher than 10% the item is disputable.

Quality of items was assessed by the absence of saturation for each of them: ceiling or floor effect and by the absence of redundancy between items evaluated by the nonparametric Spearman rank correlation coefficient (with its 95% confidence interval, CI 95%) [17]. Spearman correlations above 0.9, between 0.7 and 0.9, between 0.5 and 0.7, between 0.3 and 0.5 and below 0.3 were considered as excellent, good, moderate, poor, and negligible.

Unidimensionality of the scale was assessed using the Mokken Scale Procedure (MSP) [18]. The MSP aims at automatically partitioning the items into one or several sets by defining the dimensions of the scale and possibly a set of unscalable items using Loevinger H coefficients.

Internal consistency was estimated using the Cronbach’s α coefficient (CI 95%). A value of the score over 0.7 was considered reliable. The step-by-step Cronbach-α backward procedure was used also to check the unidimensionality of the scale.

Temporal stability was assessed to control that the scale remained stable when clinical condition did not change. Test-retest reliability was assessed between D1 and D7. Stability of the global score was assessed using the intraclass correlation coefficient (ICC; CI 95%). ICC is considered as excellent over 0.9, acceptable over 0.8, weak over 0.6 and inexistent below. The stability of each item was assessed by the weighted kappa coefficient (K; CI 95%). K is excellent over 0.8, good over 0.6, medium over 0.4, poor over 0.2, bad over 0, inexistent below 0. The Bland & Altman graph method was used to evaluate the presence of a bias [19].

Responsiveness was assessed between D7 and D30 with the Cohen’s adjusted Standardised Response Mean (SRMa) [20] and tested using the Wilcoxon paired test. SRMa > 0.8, > 0.5, > 0.2 and below are considered large, moderate, small and trivial, respectively.

Construct validity was assessed by searching convergence and divergence with other dimensions assessed by other scales. Following the convergent hypothesis, a low BBQ (low knowledge about LBP) was expected to match with high FABQ or TAMPA scores (high fear leading to movement and activities avoidance) and high Quebec or Dallas scores (high disability). On the other hand, in a divergent hypothesis, the BBQ would likely not be correlated with HAD (anxiety or depression) and VAS (pain). This validity was measured using the nonparametric Spearman rank correlation coefficient (CI 95%).

Supplementary analyses: A parametric item response theory (IRT) model was used to characterise the BBQ scale properties such as difficulty and discrimination of each item [21]. All the analyses were performed using SAS software version 9.4 (SAS Institute Inc., Cary, NC).

## Results

### Translation

The French version of the BBQ (S1 Appendix) was not different in structure from the original version. No cultural adaptation was necessary and only minor adaptations were made. The final version was sent to one of the authors of the original BBQ (A.K. Burton) who gave a feedback with the translated version and confirmed that the translated version and the English version explored the same dimension.

### Population

**Fig 1:**
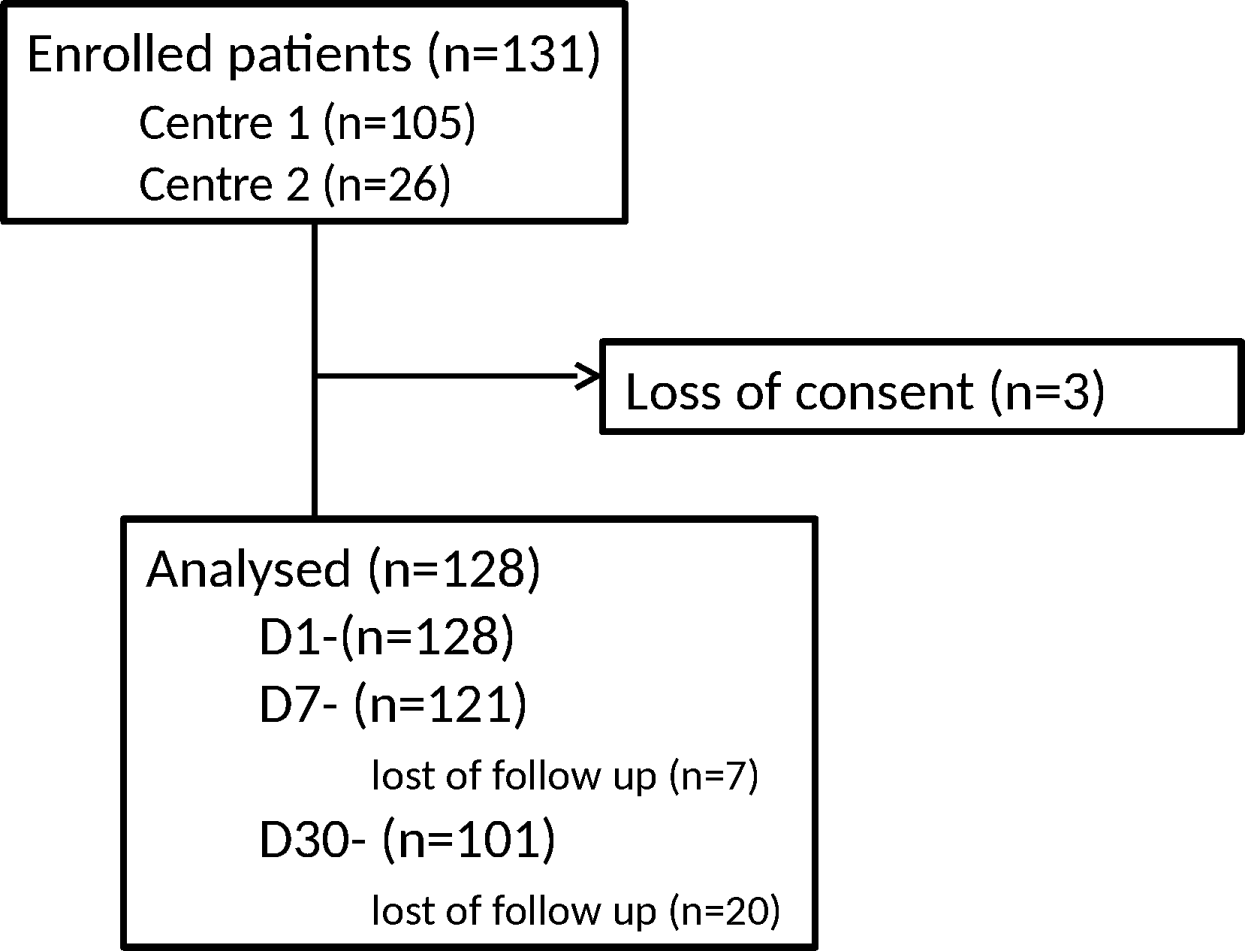
Flow chart.

Overall 131 patients were enrolled in the study: 128 patients at D1 (Centre 1, 105; Centre 2, 23), 121 at D7 (103/18) and 101 at D30 (96/5) (Fig 1). Mean age at inclusion was 43.6 ± 10.1 (range 23–68), 62 (48%) were female, median duration of LBP was 49 months (range 3–400; inter-quartile range 18–133). LBP population’ characteristics are detailed in Table 1.

**Table 1:** LBP characteristics of the study population at the reference visit (D1).

	Missing values	Median	Range	IQR
Pain (0–100)	15	44	0–100	35–52
FABQ (0–66)	1	39	4–66	26–51.5
○ Physical activity (0–24)	0	15	0–24	11–18
○ Work (0–42)	1	26.5	0–42	13.5–35.5
Tampa (0–68)	5	45	21–66	38–49
Quebec (0–100)	1	36	3–82	23.5–52
Dallas (%)				
○ Daily activities	0	55.2	22.2–84.6	46.2–64.8
○ Work-Leisure activities	1	54.5	7–94	40–67
○ Anxiety-Depression	0	40	0–100	25–60
○ Social interest	0	34	0–87	14–54
BBQ (0–45)	0	24	10–41	19–28
HAD				
○ Anxiety (0–21)	2	10	3–21	7–13
○ Depression (0–21)	2	7	1–18	4–10

### Testing the questionnaire

Regarding acceptability for the BBQ tests, most of the BBQ collected were completed and only 0.43% of all items were not filled (21/4858). A moderate ceiling effect was found for the items Q2 (32%), Q3 (36%) and Q6 (54%) as most of patients scored highest level and a floor effect for the item Q8 (60%) as most of patients did not believed that they would be, one day, forced to use a wheelchair because of their back pain. Nor floor neither ceiling effect was detected for the global score. Correlations between items were very low or absent (Spearman < 0.5) showing no redundancy between items.

One unique dimension was defined by the MSP confirming the unidimentionality of the BBQ scale. However, two items (Q1 and Q8) did not satisfy the cut-offs for the Loevinger H coefficient and were not selected by the MSP in the unique dimension of the scale.

**Fig 2:**
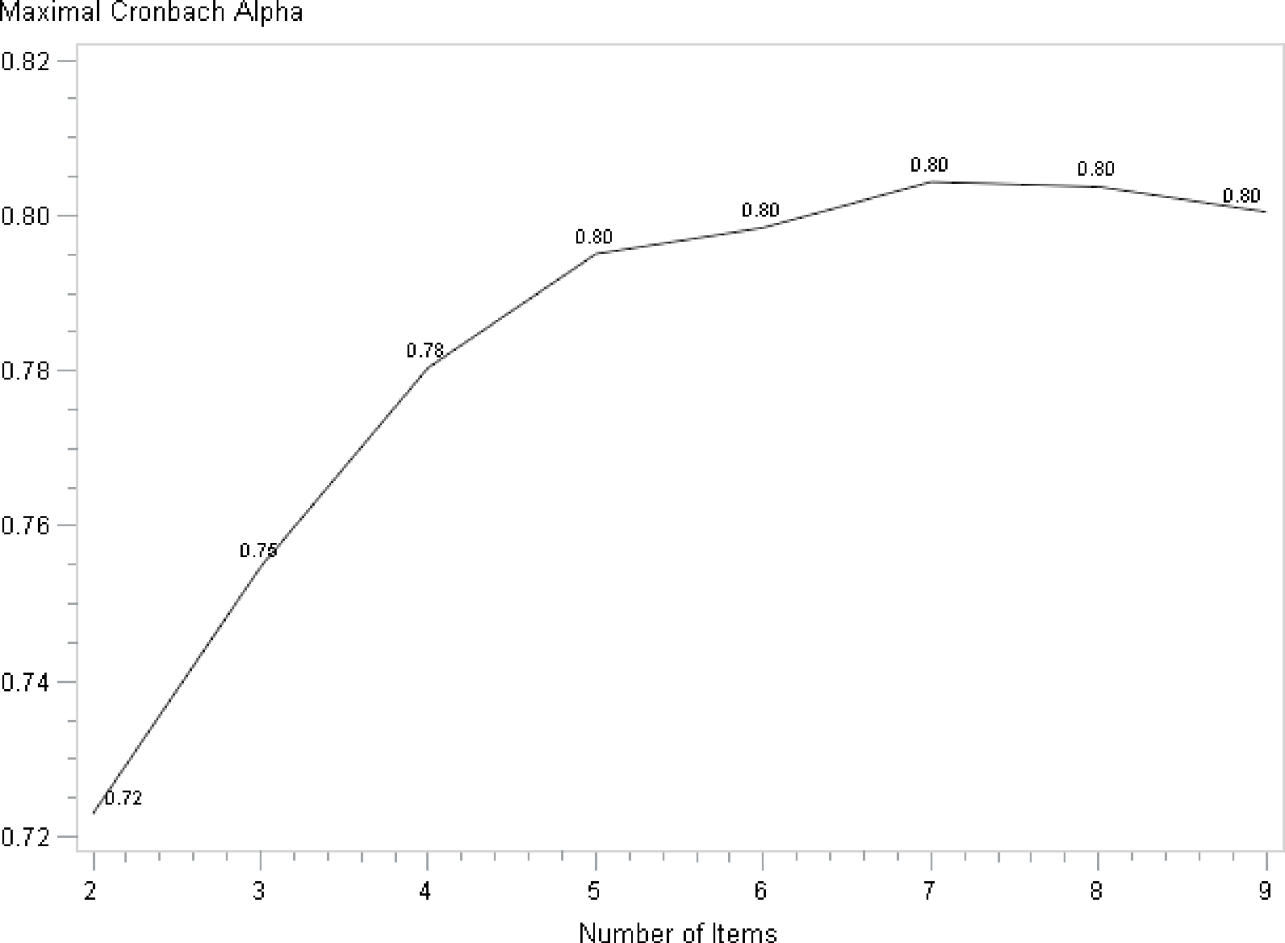
Step-by-step Cronbach α backward procedure according the number of items. The items were successively removed according the following order: Q8, Q1, Q13, Q12, Q3, Q10, Q2 (remaining Q6 and Q14).

Global Cronbach α coefficient was 0.8 (0.7–0.8) and above the 0.7 cut-off for reliable internal consistency. The step-by-step Cronbach α backward procedure confirmed the general agreement between items measuring the same construct (Fig 2). The graphical representation obtained at the end of the Cronbach α procedure is monotone increasing, which reflects a good reliability of all items. The BBQ does not have any items causing a decrease of the curve. This confirms the results from the factor analysis for unidimensionality.

**Fig 3:**
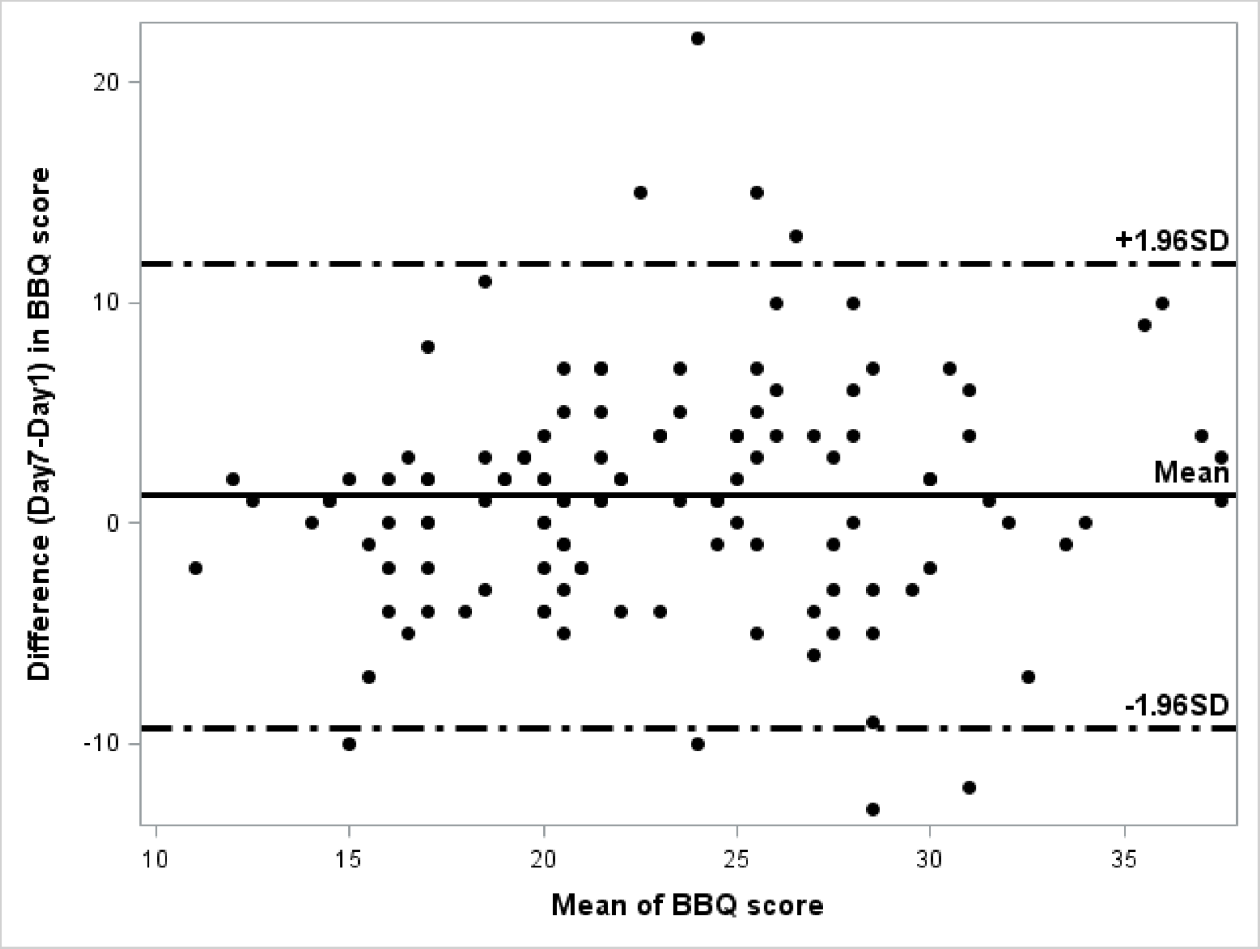
Bland & Altman method representation of a bias in the test-retest reliability method to assess temporal stability between D1 and D7. A bias between the mean differences can be detected. Here the score calculated at the second visit is +1.24 higher.

Temporal stability was low between measures performed at D1 and D7 with a 0.64 ICC (CI 95%; 0.52–0.73) for the global scale and a K varying from 0.24 (0.11–0.38) to 0.46 (0.35–0.57) for each item. The Bland & Altman graph (reported in Fig 3) shows the existence of a bias of +1.24 meaning an improvement of the beliefs already at D7. BBQ scores at D1 and D7 were 21 [18–27] (median, IQR) and 24 [19–28] respectively (p = 0.01). No difference was found between centre, gender and back pain duration between visits to explain this poor temporal stability. A change in FABQ between D1 and D7 was also found (44 [28–54] and 39 [26–51], respectively (p = 0.03)) whereas Tampa scores were not different (p = 0.87). Therefore, both BBQ and FABQ scale’s score changed significantly for the same subset of patients.

The responsiveness to the BBQ was moderate and coherent with the other scores, as shown in Table 2.

**Table 2:** Responsiveness of the BBQ compared to different scores before and after the rehabilitation procedure.

	n	Before rehabilitation Median [IQR]	After rehabilitation Median [IQR]	p	Cohen’s adjusted SRM
FABQ total (/66)	96	39.0 [26.6–50.5]	28.0 [14.5–42.0]	<0.0001	0.81
Physical Activity (/24)		16.0 [12.0–19.0]	7.0 [2.0–13.0]	<0.0001	
Work (/42)		25.5 [13.0–35.5]	19.5 [8.5–34.0]	<0.0001	
Tampa (/68)	92	45.0 [38.5–50.0]	36.0 [30.0–43.0]	<0.0001	0.98
Quebec (/100)	98	34.0 [22.0–48.0]	20.5 [12.0–38.0]	<0.0001	0.94
BBQ (/45)	100	23.5 [18.5–28.0]	27 [22.5–32.5]	<0.0001	-0.7

(IQR: inter-quartile range)

The construct validity was estimated at D7 for all variables. Consistently with the divergent hypothesis, there was no correlation with pain (r = -0.15, p = 0.19). Regarding the convergent hypothesis, the BBQ was best correlated with the Tampa (r = -0.66, p<0.001) and the FABQ (r = -0.52, p<0.001). Correlations with disability scales were poor (Quebec, r = -0.31, p<0.001; Dallas, r = -0.24 to -0.43, p<0.01). Oppositely to the divergent hypothesis, the BBQ scale was correlated, although weakly, with HADs depression (r = -0.42, p<0.001) or anxiety (r = -0.28, p = 0.0017).

**Fig 4:**
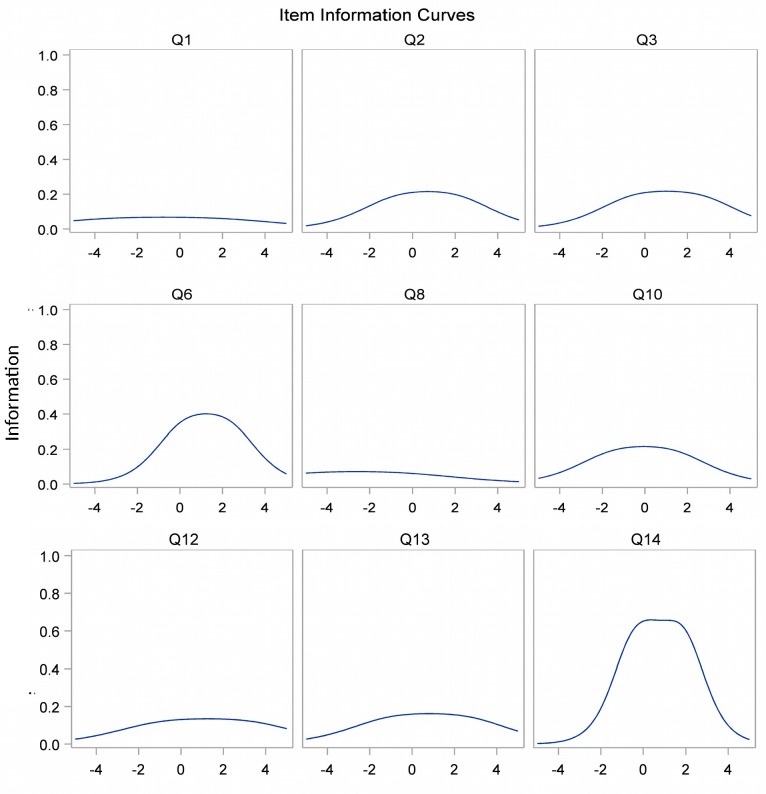
Item information curves of the nine items used for scoring the BBQ scale obtained by the parametric IRT model. Items 1, 8, 12 and 13 have a low power of information over the entire scale. These items contributed very little to the ranking of individuals. Conversely, the strongest informations power were observed for items 6 and 14. The minimal anonymized data set of the present study is available in S2 Appendix.

According to the parametric IRT model results, the most discriminative items of the scale were the items 14 and 6, and the less discriminative the items 1 and 8, which were indeed the less difficult items. The information curves obtained by the parametric IRT model are presented in Fig 4.

## Discussion

Overall, this study shows that the French version of the BBQ has good psychometric properties and can be used for evaluation of thoughts, knowledge and beliefs in patients with LBP. The translation process required only vocabulary changes with a questionnaire easily comprehensible and well accepted by patients. This aspect of low back pain is very important to address since the fear avoidance model widely explains how false beliefs and wrong thoughts contribute to wrong outcome [22].

### What the study adds

In addition to the extension of the BBQ use in French, this study brings additional knowledge. Indeed, although the construct validity, internal consistency or temporal stability have already been explored, characteristics such as quality of items and responsiveness have not been previously analysed. However, populations enrolled in previous studies were not necessarily involved in long term disability related to low back pain, usually screened for rehabilitation and education multidisciplinary programs. The population targeted in the present study gives a more accurate picture of LBP patients with severe disability related to beliefs.

Overall, the BBQ is well accepted (0.43% of no-responders) with no floor or ceiling effect detectable for the global score, neither redundancy between items (weak to very weak correlation between items, spearman < 0.5). This can explain the low rate of non-responders (14.3% for Bostick *et al*.) [23] or missing data (13.5% for Symonds *et al*.)6 described elsewhere. However, researchers should pay attention to items’ ceiling effect (Q2, Q3 and Q6), resulting in a poor discriminating ability to detect some specific LBP patients’ beliefs as most of them consider that back pain will mean pain for the rest of their life (Q3), will limit daily life activities (Q2) or work to some individual extend (Q6). Conversely, Q8 demonstrated a floor effect as most patients do not belief that back pain will lead to severe impairment. However, this thought, usually reported in educational assessment, is probably used by patients to test physicians’ opinion but cannot objectively be considered as a false belief. As a consequence, the interpretation of such item factor components must be careful.

The correlation between the nine items was good (Cronbach’s coefficient = 0.8; Fig 2) as found by others [6,23] confirming the good internal consistency. Each item seems to evaluate the same construct with α coefficient always over 0.7 and still stable after each deletion without any significant redundancy between items.

### Comparison to other scores

The concept that fear and beliefs lead to movement and activities avoidance, disability, and therefore inevitable consequences of low back pain, according to the fear avoidance model [24] was believable. As expected, the BBQ was convergent with others scales which have close concepts such as Tampa for kinesiophobia and FABQ for fear avoidance of work and physical activity (spearman coefficient >0.5; p<0.001). This association was higher than regarding functional scales: for example, the relationship with the Quebec scale was weaker but still significant (spearman coefficient <0.5; p<0.001).

Unexpectedly, disability and depression, explored with Dallas and HAD scales were convergent with BBQ although weakly (spearman coefficient <0.5; p<0.01). However, the link between HAD (anxiety part) and Tampa scale [25] or FABQ and HAD [12,26] can explain such a relation.

Therefore, the BBQ gives clinicians some new information that are probably not provided elsewhere considering the weak or inexistent correlations observed with other scores.

In a previous study, Bostick *et al*. [23] have tested the relationship between beliefs and history of low back pain. However, the results suggested that beliefs were not exclusively linked to pain. This is confirmed by the divergence of BBQ with pain evaluation. Fear can lead to pain (Montaigne; “He who fears shall suffer, already suffers what he fears”) but does not explain or encompass pain. Finally, the best correlations observed with Tampa and FABQ were expected since fear of movement and avoidance are conceptually close [26–28]. Therefore, in a non-worker LBP population, the BBQ can be easily used and interpreted in this frame.

Finally, for the first time the responsiveness of the BBQ after an educational and rehabilitation program has been tested. In most cases, beliefs improve after multidisciplinary interventions and accordingly the BBQ score increases after one week of educational intervention (4.5 points increasing; p<0.0001). The BBQ score can therefore be used as an objective tool of education assessment in CLBP. Furthermore, associated with a rehabilitation intervention, the BBQ was able to detect changes accordingly with other scales testing avoidance of movement, return to work, kinesophobia or and functional abilities as reported here (Table 2).

### Limitations

Temporal reliability tests have shown that test-retest reliability between D1 and D7 was either weak or poor according to definition. This can be unexpected since no intervention was scheduled during this period. Two hypotheses may explain this result. First, it could be suspected that the BBQ is not stable with time and probably variable whereas the clinical status is stable; however, this hypothesis requires confirmation that no clinical change has occurred. Second, it can be suspected that the patient educational assessment proposed at inclusion (D1) has already modified the related beliefs. The changes observed on the FABQ scores between D1 and D7 pleads this reason. Moreover, FABQ have demonstrated a good temporal stability [26, 27]. Since educational interventions modify beliefs in LBP population [29–31], changes in the BBQ and another score would rather support the sensitivity to change of BBQ. In this study, patients, were enrolled because of chronic LBP and were evaluated the same day of the inclusion. It is possible therefore that the educational questioning provided the first day would have led to changes in back pain thoughts because of the discussions with the team and/or exchanges between participants. This hypothesis however, needs to be confirmed by studies with no educational assessment.

Finally, data on the professional status of the patients included was not specified so we are not able to extrapolate our results to the general LBP population.

### Perspectives

The use of the BBQ score for evaluation of inevitable consequences of beliefs related to LBP and educational assessment seems of interest. However, the usefulness of the whole set of items could be questioned. Bostick and colleagues [23] have already underlined the little change in the overall score whether Q1 remained or not (correlation still very high and reliability unchanged). Similarly, in the present study, the interest of Q1 as well as Q8 were disputable. Indeed, these two items were not selected in the MSP, they demonstrated the weakest input for internal consistency and they were the less discriminative item in the IRT model. Therefore, it could be interesting to test the validity of the BBQ using only “loading items” for scoring and propose another form of the questionnaire. Nevertheless, the distractors are interesting in an educational perspective. Indeed, the BBQ is typically oriented toward emotions, thoughts and beliefs assessment and can be easily used as educational support. Each item explores most of the questions asked by LBP patients (e.g. “*back trouble will stop you from working”* or *“means you end up in a wheelchair”)* and can open a face-to-face discussion on individual worst beliefs. In this perspective, the BBQ may enhance their ability to catch unpleasant but still vague related thoughts for a better understanding and management [7,24].

## Conclusion

The BBQ, now available in French language, showed good psychometric properties to assess false beliefs and related fear in French LBP populations. These results suggest that the questionnaire can be used either for evaluation in international trials or as a part of self-care training.

## Supporting information

S1 Appendix The French version of the Back Belief Questionnaire. (TIFF)

S2 Appendix Minimal anonymized data set used for BBQ validation. (PDF)

## References

[1] Waddell G. 1987 Volvo award in clinical sciences. A new clinical model for the treatment of low-back pain. Spine. 1987;12: 632–44. 2961080 10.1097/00007632-198709000-00002

[2] Rainville J, Smeets RJEM, Bendix T, Tveito TH, Poiraudeau S, Indahl AJ. Fear-avoidance beliefs and pain avoidance in low back pain—translating research into clinical practice. Spine J Off J North Am Spine Soc. 2011;11: 895–903. 10.1016/j.spinee.2011.08.006 21907633

[3] Coudeyre E, Tubach F, Rannou F, Baron G, Coriat F, Brin S, et al Fear-avoidance beliefs about back pain in patients with acute LBP. Clin J Pain. 2007;23: 720–5. doi: 10.1097/AJP.0b013e31814da407 17885352 10.1097/AJP.0b013e31814da407

[4] Coudeyre E, Rannou F, Tubach F, Baron G, Coriat F, Brin S, et al General practitioners’ fear-avoidance beliefs influence their management of patients with low back pain. Pain. 2006;124: 330–7. doi: 10.1016/j.pain.2006.05.003 16750297 10.1016/j.pain.2006.05.003

[5] Wertli MM, Rasmussen-Barr E, Held U, Weiser S, Bachmann LM, Brunner F. Fear-avoidance beliefs-a moderator of treatment efficacy in patients with low back pain: a systematic review. Spine J Off J North Am Spine Soc. 2014;14: 2658–78. 10.1016/j.spinee.2014.02.033 24614254

[6] Symonds TL, Burton AK, Tillotson KM, Main CJ. Do attitudes and beliefs influence work loss due to low back trouble? Occup Med Oxf Engl. 1996;46: 25–32. 10.1093/occmed/46.1.25 8672790

[7] Dupeyron A, Ribinik P, Gélis A, Genty M, Claus D, Hérisson C, et al Education in the management of low back pain: literature review and recall of key recommendations for practice. Ann Phys Rehabil Med. 2011;54: 319–35. doi: 10.1016/j.rehab.2011.06.001 21782541 10.1016/j.rehab.2011.06.001

[8] Beaton DE, Bombardier C, Guillemin F, Ferraz MB. Guidelines for the process of cross-cultural adaptation of self-report measures. Spine (Phila Pa 1976). 2000;25: 3186–91. 11124735 10.1097/00007632-200012150-00014

[9] Fermanian J. [Validation of assessment scales in physical medicine and rehabilitation: how are psychometric properties determined?]. Ann Readapt Med Phys. 2005;48: 281–7. doi: 10.1016/j.annrmp.2005.04.004 15923054 10.1016/j.annrmp.2005.04.004

[10] Guillemin F, Bombardier C, Beaton D. Cross-cultural adaptation of health-related quality of life measures: literature review and proposed guidelines. J Clin Epidemiol. 1993;46: 1417–32. 8263569 10.1016/0895-4356(93)90142-n

[11] Waddell G, Newton M, Henderson I, Somerville D, Main CJ. A Fear-Avoidance Beliefs Questionnaire (FABQ) and the role of fear-avoidance beliefs in chronic low back pain and disability. Pain. 1993;52: 157–68 8455963 10.1016/0304-3959(93)90127-B

[12] Chaory K, Fayad F, Rannou F, Lefèvre-Colau MM, Fermanian J, Revel M, Poiraudeau S. Validation of the French version of the fear avoidance belief questionnaire. Spine (Phila Pa 1976). 2004;29: 908–13. 15082995 10.1097/00007632-200404150-00018

[13] Kopec JA, Esdaile JM, Abrahamowicz M, Abenhaim L, Wood-Dauphinee S, Lamping DL, et al The Quebec Back Pain Disability Scale. Measurement properties. Spine. 1995 2 1;20: 341–52. 7732471 10.1097/00007632-199502000-00016

[14] Zigmond AS, Snaith RP. The hospital anxiety and depression scale. Acta Psychiatr Scand. 1983 6;67: 361–70. 6880820 10.1111/j.1600-0447.1983.tb09716.x

[15] Kori SH, Miller RP, Todd DD. Kinesiophobia: a new view of chronic pain behavior. Pain Manag. 1990;3: 35–43.

[16] Lawlis GF, Cuencas R, Selby D, McCoy CE. The development of the Dallas Pain Questionnaire. An assessment of the impact of spinal pain on behavior. Spine. 1989;14: 511–6. 2524890 10.1097/00007632-198905000-00007

[17] Hinkle DE, Wiersma WW, Jurs SG. Applied statistics for the behavioral sciences. 2003

[18] Hemker BT, Sijtsma K, Molenaar IW. Selection of Unidimensional Scales from a Multidimensional Item Bank in the Polytomous Mokken IRT Model. Applied Psychological Measurement;19: 337–52

[19] Bland JM, Altman DG. Comparing methods of measurement: why plotting difference against standard method is misleading. Lancet. 1995;346: 1085–7 7564793 10.1016/s0140-6736(95)91748-9

[20] Middel B, van Sonderen E. Statistical significant change versus relevant or important change in (quasi) experimental design: some conceptual and methodological problems in estimating magnitude of intervention-related change in health services research. Int J Integr Care. 2002;2: e15 16896390 10.5334/ijic.65 PMC1480399

[21] de Boeck P, Wilson M. Explanatory item response models. A generalized linear and nonlinear approach New York, New Jersey: Springer 2004.

[22] Feuerstein M, Beattie P. Biobehavioral factors affecting pain and disability in low back pain: mechanisms and assessment. Phys Ther. 1995;75: 267–80. 7899485 10.1093/ptj/75.4.267

[23] Bostick GP, Schopflocher D, Gross DP. Validity evidence for the back beliefs questionnaire in the general population. Eur J Pain. 2013;17: 1074–81 doi: 10.1002/j.1532-2149.2012.00275.x 23335330 10.1002/j.1532-2149.2012.00275.x

[24] Brox JI. Current evidence on catastrophizing and fear avoidance beliefs in low back pain patients. Spine J. 2014;14: 2679–81. doi: 10.1016/j.spinee.2014.08.454 25441973 10.1016/j.spinee.2014.08.454

[25] Thomas EN, Pers YM, Mercier G, Cambiere JP, Frasson N, Ster F, Hérisson C, Blotman F. The importance of fear, beliefs, catastrophizing and kinesiophobia in chronic low back pain rehabilitation. Ann Phys Rehabil Med. 2010;53: 3–14. doi: 10.1016/j.rehab.2009.11.002 20022577 10.1016/j.rehab.2009.11.002

[26] Waddell G, Newton M, Henderson I, Somerville D, Main CJ. A Fear-Avoidance Beliefs Questionnaire (FABQ) and the role of fear-avoidance beliefs in chronic low back pain and disability. Pain. 1993;52:157–68. 8455963 10.1016/0304-3959(93)90127-B

[27] Swinkels-Meewisse EJ, Swinkels RA, Verbeek AL, Vlaeyen JW, Oostendorp RA. Psychometric properties of the Tampa Scale for kinesiophobia and the fear-avoidance beliefs questionnaire in acute low back pain. Man Ther. 2003;8: 29–36. 12586559 10.1054/math.2002.0484

[28] Woby SR, Roach NK, Urmston M, Watson PJ. Psychometric properties of the TSK-11: a shortened version of the Tampa Scale for Kinesiophobia. Pain. 2005;117: 137–44. doi: 10.1016/j.pain.2005.05.029 16055269 10.1016/j.pain.2005.05.029

[29] Buchbinder R, Jolley D, Wyatt M. 2001 Volvo award winner in clinical studies: effects of a media campaign on back pain beliefs and its potential influence on management of low back pain in general practice. Spine 2001;26: 2535–42 11725233 10.1097/00007632-200112010-00005

[30] Burton AK, Waddell G, Tillotson KM, Summerton N. Information and advice to patients with back pain can have a positive effect. A randomized controlled trial of a novel educational booklet in primary care. Spine (Phila Pa 1976). 1999;24: 2484–91 10626311 10.1097/00007632-199912010-00010

[31] Tavares Figueiredo I, Dupeyron A, Tran B, Duflos C, Julia M, Hérisson C, Coudeyre E. Educational self-care objectives within a funstional spine restoration program. Retrospective study of 104 patients. Ann Phys Rehabil Med. 2016 12;59(5–6):289–293. doi: 10.1016/j.rehab.2016.03.006 27157543 10.1016/j.rehab.2016.03.006

