# Supplementary figures and images for: The Back Belief Questionnaire is efficient to assess false beliefs and related fear in low back pain populations: A transcultural adaptation and validation study
Source: PLoS One. 2017 Dec 6;12(12):e0186753. doi: 10.1371/journal.pone.0186753 (PMC5718465; doi:10.1371/journal.pone.0186753)

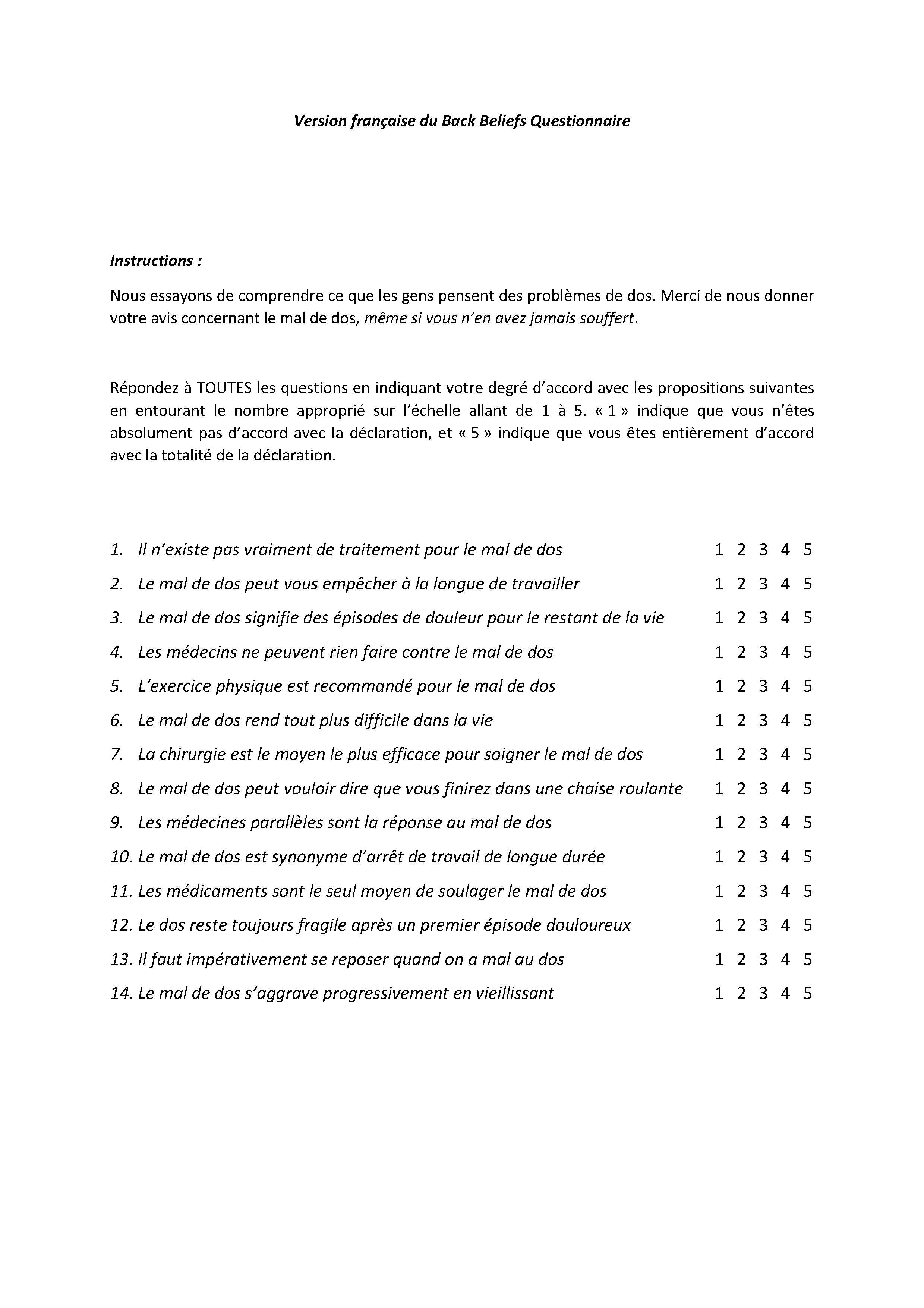

Supplement: S1 Appendix — (TIFF) [file pone.0186753.s001.tiff]
