# Supplementary material for: The Back Belief Questionnaire is efficient to assess false beliefs and related fear in low back pain populations: A transcultural adaptation and validation study
Source: PLoS One. 2017 Dec 6;12(12):e0186753. doi: 10.1371/journal.pone.0186753 (PMC5718465; doi:10.1371/journal.pone.0186753)
Supplement: S2 Appendix — (PDF) [file pone.0186753.s002.pdf]

id	centre	gender	age	lbp_duration	d1_bbq_coll	d1_bbq_comp	d1_bbq_nfill	d1_bbq_q1
C01P001	C01	Female	39	72	Yes	Yes	1	4
C01P002	C01	Female	34	6	Yes	Yes	0	2
C01P003	C01	Male	50	312	Yes	Yes	0	5
C01P004	C01	Male	32	13	Yes	Yes	0	5
C01P005	C01	Male	53	47	Yes	Yes	0	4
C01P006	C01	Female	50	133	Yes	Yes	0	4
C01P007	C01	Female	48	47	Yes	Yes	0	1
C01P008	C01	Female	42	23	Yes	Yes	0	2
C01P009	C01	Female	61	18	Yes	Yes	0	3
C01P010	C01	Male	46	77	Yes	Yes	0	3
C01P011	C01	Male	54	12	Yes	Yes	0	5
C01P012	C01	Male	57	180	Yes	Yes	0	1
C01P013	C01	Female	42	8	Yes	Yes	0	2
C01P014	C01	Male	29	120	Yes	Yes	0	2
C01P015	C01	Male	50	3	Yes	Yes	0	4
C01P016	C01	Female	60	85	Yes	Yes	0	4
C01P017	C01	Female	39	86	Yes	Yes	0	2
C01P018	C01	Female	29	62	No	No	14	.
C01P019	C01	Female	32	2	Yes	Yes	0	4
C01P020	C01	Female	50	110	Yes	Yes	0	2
C01P021	C01	Female	54	7	No	No	14	.
C01P022	C01	Female	51	362	Yes	Yes	0	3
C01P023	C01	Male	53	210	Yes	Yes	0	2
C01P024	C01	Female	51	18	Yes	Yes	0	5
C01P025	C01	Male	57	234	Yes	Yes	0	5
C01P026	C01	Male	43	271	Yes	Yes	0	1
C01P027	C01	Female	46	247	Yes	Yes	0	3
C01P028	C01	Female	42	7	Yes	Yes	0	3
C01P029	C01	Female	37	199	Yes	Yes	0	1
C01P030	C01	Male	33	8	Yes	Yes	0	3
C01P031	C01	Female	41	188	Yes	Yes	0	1
C01P032	C01	Female	49	18	Yes	Yes	1	3
C01P033	C01	Female	50	176	Yes	Yes	0	4
C01P034	C01	Male	47	56	Yes	Yes	0	3
C01P035	C01	Female	51	105	Yes	Yes	0	4
C01P036	C01	Male	52	13	Yes	Yes	0	3
C01P037	C01	Male	25	8	Yes	Yes	0	3
C01P038	C01	Female	50	81	Yes	Yes	0	2
C01P039	C01	Female	57	117	Yes	Yes	0	2
C01P040	C01	Male	53	18	Yes	Yes	0	3
C01P041	C01	Male	50	22	Yes	Yes	0	3
C01P042	C01	Male	34	22	Yes	Yes	0	3
C01P043	C01	Male	44	202	Yes	Yes	0	3
C01P044	C01	Female	68	214	Yes	Yes	0	4
C01P045	C01	Male	34	94	Yes	Yes	0	1
C01P046	C01	Female	34	34	Yes	Yes	0	2
C01P047	C01	Male	27	8	Yes	Yes	0	3
C01P048	C01	Male	31	180	Yes	Yes	0	2

C01P049	C01	Female	30	24	Yes	Yes	0	5
C01P050	C01	Male	35	14	Yes	Yes	0	1
C01P051	C01	Female	25	17	Yes	Yes	0	2
C01P052	C01	Male	56	121	Yes	Yes	0	2
C01P053	C01	Male	33	8	Yes	Yes	0	4
C01P054	C01	Female	61	121	Yes	Yes	0	3
C01P055	C01	Female	46	19	Yes	Yes	0	3
C01P056	C01	Male	56	11	Yes	Yes	0	4
C01P057	C01	Female	49	27	Yes	Yes	0	4
C01P058	C01	Male	53	219	Yes	Yes	0	1
C01P059	C01	Male	40	12	Yes	Yes	0	1
C01P060	C01	Male	67	400	Yes	Yes	0	3
C01P061	C01	Male	44	196	Yes	Yes	0	3
C01P062	C01	Female	42	12	Yes	Yes	0	3
C01P063	C01	Male	34	12	Yes	Yes	0	1
C01P064	C01	Male	31	28	Yes	Yes	1	3
C01P065	C01	Female	37	15	Yes	Yes	0	4
C01P066	C01	Female	44	125	Yes	Yes	0	4
C01P067	C01	Male	31	29	Yes	Yes	0	1
C01P068	C01	Male	39	17	Yes	Yes	1	3
C01P069	C01	Female	39	53	Yes	Yes	0	5
C01P070	C01	Male	31	18	Yes	Yes	0	1
C01P071	C01	Female	36	19	Yes	Yes	0	4
C01P072	C01	Male	50	42	Yes	Yes	0	1
C01P073	C01	Male	45	378	Yes	Yes	1	3
C01P074	C01	Female	50	30	Yes	Yes	0	1
C01P075	C01	Male	33	67	Yes	Yes	0	4
C01P076	C01	Male	28	19	Yes	Yes	0	4
C01P077	C01	Female	56	55	Yes	Yes	0	5
C01P078	C01	Male	37	53	Yes	Yes	0	2
C01P079	C01	Male	32	4	Yes	Yes	0	5
C01P080	C01	Male	36	80	Yes	Yes	0	4
C01P081	C01	Female	57	119	Yes	Yes	0	3
C01P082	C01	Female	44	56	Yes	Yes	0	4
C01P083	C01	Male	42	10	Yes	Yes	0	3
C01P084	C01	Male	56	344	Yes	Yes	0	3
C01P085	C01	Female	34	141	Yes	Yes	0	3
C01P086	C01	Male	30	15	Yes	Yes	0	3
C01P087	C01	Female	55	369	Yes	Yes	1	3
C01P088	C01	Female	54	129	Yes	Yes	0	2
C01P089	C01	Female	53	7	Yes	Yes	0	4
C01P090	C01	Female	32	19	Yes	Yes	0	1
C01P091	C01	Male	25	11	Yes	Yes	0	3
C01P092	C01	Female	26	178	Yes	Yes	0	5
C01P093	C01	Male	42	49	Yes	Yes	0	1
C01P094	C01	Male	58	34	Yes	Yes	0	3
C01P095	C01	Female	37	203	Yes	Yes	0	4
C01P096	C01	Female	42	20	Yes	Yes	0	3
C01P097	C01	Female	44	28	Yes	Yes	0	4
C01P098	C01	Male	41	36	Yes	Yes	0	3

C01P099	C01	Female	45	71	Yes	Yes	0	2
C01P100	C01	Female	62	71	Yes	Yes	0	4
C01P101	C01	Male	57	47	Yes	Yes	0	1
C01P102	C01	Female	47	28	Yes	Yes	0	3
C01P103	C01	Male	32	72	Yes	Yes	0	1
C01P104	C01	Male	55	138	Yes	Yes	0	3
C01P105	C01	Male	48	168	Yes	Yes	0	5
C02P002	C02	Male	39	.	Yes	Yes	0	3
C02P003	C02	Male	54	217	Yes	Yes	0	4
C02P004	C02	Female	51	43	Yes	Yes	0	5
C02P005	C02	Female	56	105	Yes	Yes	0	2
C02P006	C02	Female	43	26	Yes	Yes	10	.
C02P007	C02	Male	43	45	Yes	Yes	0	4
C02P008	C02	Male	55	183	Yes	Yes	0	4
C02P009	C02	Male	37	73	Yes	Yes	0	5
C02P010	C02	Male	23	14	Yes	Yes	0	5
C02P011	C02	Female	24	11	Yes	Yes	0	2
C02P012	C02	Male	45	204	Yes	Yes	0	3
C02P013	C02	Female	38	.	Yes	Yes	0	2
C02P014	C02	Male	36	.	Yes	Yes	0	2
C02P015	C02	Female	42	49	Yes	Yes	0	3
C02P016	C02	Male	49	.	Yes	Yes	0	4
C02P017	C02	Male	34	.	Yes	Yes	0	1
C02P018	C02	Male	38	313	Yes	Yes	0	3
C02P019	C02	Male	44	.	Yes	Yes	1	1
C02P020	C02	Female	43	271	Yes	Yes	0	3
C02P021	C02	Female	48	.	Yes	Yes	0	3
C02P023	C02	Female	53	49	Yes	Yes	0	4
C02P025	C02	Male	49	31	Yes	Yes	0	1
C02P026	C02	Female	34	224	Yes	Yes	0	2

d1_bbq_q2	d1_bbq_q3	d1_bbq_q4	d1_bbq_q5	d1_bbq_q6	d1_bbq_q7	d1_bbq_q8	d1_bbq_q9
5	5	4	5	5	1	3	.
5	1	1	5	5	1	1	4
5	5	3	4	4	2	4	2
5	5	4	4	5	1	3	5
5	5	3	3	5	3	2	1
5	4	3	3	5	1	1	3
3	3	1	5	5	3	4	1
4	5	3	3	5	5	1	1
4	5	3	4	4	2	5	2
5	5	3	3	5	3	3	1
2	4	1	1	5	2	2	1
2	2	1	3	3	2	1	1
5	5	5	5	5	2	3	1
4	5	2	4	4	3	3	2
4	4	3	4	3	1	1	3
5	5	2	4	5	3	1	3
5	3	1	4	5	2	2	3
.	.	.	.	.	.	.	.
5	5	1	5	5	2	1	2
3	5	4	5	4	2	1	2
.	.	.	.	.	.	.	.
5	4	4	3	5	3	4	4
5	2	1	5	5	1	1	3
1	1	5	1	1	5	1	1
4	5	4	3	5	2	1	1
5	5	1	5	5	5	1	1
5	5	1	3	5	3	3	3
5	4	3	5	5	1	1	4
5	5	1	5	5	1	3	5
3	2	2	3	5	2	1	1
4	3	4	5	3	1	1	3
5	5	5	5	5	1	1	.
2	4	2	5	3	1	4	2
5	5	3	3	5	3	2	1
4	5	4	5	5	1	1	1
5	3	2	3	5	1	1	3
3	5	2	4	5	3	2	3
3	3	2	5	5	3	1	2
5	5	3	3	5	2	2	3
3	5	3	2	4	2	2	3
4	2	1	1	2	4	1	3
4	4	3	4	5	2	4	4
5	5	3	5	3	2	3	3
1	3	2	5	1	1	5	5
2	3	1	5	5	1	1	3
5	5	2	5	3	3	4	3
3	5	3	3	5	3	3	1
5	2	1	4	2	2	1	3

4	5	5	4	5	4	5	3
5	2	2	5	5	3	2	2
2	4	1	5	4	1	1	3
3	4	2	5	5	1	1	3
5	5	3	3	5	1	1	5
4	4	3	1	4	4	1	2
3	5	4	5	5	1	1	3
3	5	4	3	4	1	1	2
5	4	3	5	5	3	3	5
3	1	1	1	4	1	1	1
5	3	1	5	5	1	3	3
4	4	3	2	2	1	3	2
2	2	3	1	1	3	5	3
1	3	1	5	4	1	1	1
5	3	1	5	5	3	1	3
5	4	4	5	5	1	1	.
4	5	4	5	5	1	1	3
5	5	4	4	5	3	1	1
3	3	1	3	2	3	1	3
5	2	3	.	5	3	1	3
5	5	1	1	5	1	1	5
4	2	1	5	3	1	1	5
4	4	5	5	5	3	1	3
5	5	1	5	5	1	1	3
4	5	3	5	5	1	1	.
1	2	1	5	2	1	1	3
4	4	4	4	4	3	3	3
4	4	3	4	4	4	2	2
5	3	5	3	5	3	1	3
3	3	2	5	3	1	1	3
4	5	3	3	5	3	3	3
5	5	4	3	5	2	3	3
5	5	3	3	5	2	1	2
3	4	2	4	3	1	1	3
3	2	4	3	3	3	1	2
3	5	2	5	4	2	1	3
4	4	2	4	5	1	1	2
5	4	3	5	5	3	1	3
5	5	3	.	3	2	1	3
5	5	3	3	3	1	1	2
5	5	4	4	5	3	4	3
5	5	1	4	5	2	2	2
4	4	3	5	5	1	3	3
1	3	1	5	1	1	1	1
5	1	1	5	5	3	1	1
4	4	3	5	5	3	1	5
4	4	2	3	4	3	2	4
4	4	4	5	3	4	4	3
5	4	5	5	5	1	1	3
5	5	3	5	3	1	1	3

5	5	3	3	5	3	1	1
5	4	4	5	4	2	2	2
5	5	3	1	5	3	3	3
3	3	3	1	1	3	3	3
3	1	1	5	3	3	1	3
5	5	3	4	5	3	3	1
1	3	5	5	5	1	1	1
5	3	2	4	4	2	3	1
5	5	4	4	5	4	4	3
3	5	5	5	5	3	3	4
2	4	2	5	5	3	3	1
.	2	.	.	1	2	.	.
5	4	4	5	5	3	4	3
5	5	4	1	5	1	3	2
3	5	3	2	4	3	3	2
4	3	3	5	5	2	5	2
4	4	2	4	4	1	1	3
1	5	4	5	3	3	1	3
3	3	1	2	5	1	1	1
4	3	5	3	5	5	3	2
5	4	3	3	5	5	3	5
3	4	3	5	5	1	1	3
1	3	1	1	3	2	1	4
4	3	3	3	3	1	3	3
5	5	1	5	5	1	1	.
5	3	3	4	5	3	1	3
5	3	3	5	5	3	5	3
5	5	4	5	5	4	1	2
3	3	1	3	5	3	1	2
1	4	2	3	5	1	1	4

d1_bbq_q10	d1_bbq_q11	d1_bbq_q12	d1_bbq_q13	d1_bbq_q14	d1_bbq	d1_fabq	d7_attend	d7_pain
5	4	5	5	5	12	32	Yes	80
2	1	3	4	4	27	41	Yes	80
4	4	4	3	4	16	57	Yes	50
1	1	3	4	5	18	59	Yes	80
1	4	3	5	5	19	59	Yes	.
4	1	2	3	5	21	43	Yes	60
3	2	5	4	5	21	41	Yes	30
3	1	1	5	5	23	.	Yes	60
2	2	5	5	5	16	.	Yes	60
5	3	5	5	5	13	59	Yes	60
4	1	3	4	5	20	51	Yes	80
1	2	2	3	3	36	39	Yes	0
3	1	4	5	4	18	47	Yes	50
4	3	4	5	5	18	55	Yes	60
3	3	5	4	5	21	39	Yes	.
5	2	5	5	5	14	61	Yes	.
3	2	5	3	4	22	20	Yes	.
.	.	.	.	.	.	.	Yes	.
3	1	4	4	4	19	56	Yes	.
1	1	4	5	4	25	63	Yes	20
.	.	.	.	.	.	33	Yes	60
3	3	3	3	4	20	47	Yes	.
3	1	5	2	5	24	39	Yes	.
1	5	1	5	1	37	.	Yes	.
3	4	5	5	5	16	20	Yes	.
5	4	5	5	5	17	46	Yes	.
3	3	5	3	5	17	19	Yes	.
4	5	5	5	5	17	14	Yes	.
5	3	5	5	5	15	57	Yes	.
3	3	2	3	3	29	47	No	.
1	1	3	3	3	32	20	Yes	.
5	2	5	5	5	15	38	Yes	.
2	1	3	4	4	24	27	Yes	.
5	3	5	5	5	14	44	Yes	.
4	3	5	4	5	17	64	No	.
3	3	4	5	5	20	51	Yes	60
4	1	5	4	4	19	55	Yes	.
2	4	3	2	4	29	22	Yes	.
5	4	4	3	5	18	53	Yes	.
3	3	3	3	4	24	48	Yes	40
3	1	4	4	2	29	44	Yes	0
4	3	3	3	4	20	41	Yes	.
4	3	4	3	4	20	17	Yes	.
1	5	2	5	1	31	7	Yes	.
2	3	3	3	3	31	28	Yes	20
2	4	2	4	5	22	39	Yes	.
1	5	5	5	4	20	58	Yes	.
3	2	3	3	4	29	54	Yes	.

5	4	5	4	5	11	57	Yes	.
1	3	5	5	5	23	26	Yes	.
1	3	4	4	4	28	46	Yes	70
3	3	5	5	5	21	25	Yes	.
5	2	4	4	5	16	63	Yes	.
1	5	1	3	5	28	21	Yes	10
2	1	3	3	5	24	49	Yes	20
1	2	3	4	4	25	29	Yes	.
3	4	4	3	5	18	60	Yes	.
1	1	1	3	2	37	43	Yes	.
3	1	5	1	5	23	40	Yes	.
2	2	2	5	4	25	25	Yes	0
1	4	2	3	5	30	46	Yes	60
1	1	3	5	5	28	.	Yes	60
3	3	5	5	5	21	57	Yes	50
5	4	3	1	3	24	46	Yes	40
4	5	1	5	2	23	54	Yes	.
5	3	3	4	4	18	65	Yes	.
3	3	3	4	4	30	27	Yes	30
5	1	3	4	4	22	24	Yes	30
5	1	5	5	5	13	48	Yes	40
2	1	2	2	3	34	20	Yes	40
1	4	4	3	5	23	44	Yes	20
1	1	5	3	3	25	51	Yes	10
1	2	4	5	5	21	27	Yes	40
1	2	5	3	2	36	4	Yes	30
1	4	3	3	4	24	30	Yes	20
4	3	5	3	4	20	48	Yes	30
1	4	5	5	5	19	26	Yes	60
1	3	1	3	3	34	22	Yes	20
3	3	4	3	4	18	64	Yes	20
5	3	5	5	5	12	65	Yes	50
5	3	3	3	3	21	40	Yes	40
1	3	3	5	2	28	35	Yes	50
3	4	3	3	4	29	41	Yes	30
2	3	4	2	4	26	21	Yes	20
2	4	5	4	3	23	26	Yes	60
5	3	4	5	5	17	54	Yes	10
3	3	5	3	5	21	26	Yes	60
4	5	4	5	5	20	54	Yes	50
4	4	4	5	5	13	58	Yes	50
4	4	5	5	2	20	63	Yes	30
3	4	4	4	5	19	51	Yes	40
1	1	5	1	5	31	18	Yes	50
3	3	3	3	1	31	38	Yes	30
3	1	3	4	4	23	44	Yes	30
4	4	3	4	4	21	44	Yes	60
4	3	4	4	5	19	.	Yes	10
4	5	4	3	4	20	46	Yes	50
3	4	3	2	4	25	37	Yes	40

3	1	1	3	4	25	36	Yes	50
2	2	4	4	3	22	.	Yes	30
3	5	1	1	1	29	32	Yes	50
3	2	2	3	2	31	43	Yes	20
3	3	5	3	3	31	30	Yes	30
4	3	5	5	5	14	60	Yes	40
1	1	5	5	2	26	37	Yes	20
4	3	4	5	5	18	51	Yes	40
3	2	2	3	5	18	44	Yes	.
5	5	5	5	5	13	.	No	.
3	3	3	5	5	22	29	Yes	.
.	.	.	2	.	.	.	No	.
3	3	5	3	3	18	53	Yes	30
3	5	5	5	5	14	53	Yes	100
3	3	4	3	3	21	51	Yes	.
1	1	5	3	3	20	32	No	.
2	2	4	4	3	26	49	Yes	70
1	1	1	3	1	35	0	Yes	10
2	2	2	3	3	30	46	Yes	.
4	5	3	4	4	22	41	Yes	.
2	4	5	5	4	18	48	Yes	.
5	2	4	3	4	21	56	Yes	30
3	3	3	2	4	33	12	Yes	50
3	1	3	5	3	24	26	No	.
5	1	5	5	5	17	60	Yes	.
1	2	4	1	2	29	17	Yes	.
3	4	3	5	4	18	66	Yes	.
5	5	5	5	4	15	60	Yes	60
3	3	2	2	3	31	31	No	.
1	3	2	1	2	35	12	Yes	.

d7_bbq_coll	d7_bbq_comp	d7_bbq_nfill	d7_bbq_q1	d7_bbq_q2	d7_bbq_q3	d7_bbq_q4	d7_bbq_q5
Yes	Yes	1	5	5	5	2	3
Yes	Yes	0	1	4	1	1	5
Yes	Yes	0	4	5	5	4	5
Yes	Yes	0	3	4	4	4	5
Yes	Yes	0	3	5	4	2	2
Yes	Yes	0	3	5	3	4	5
Yes	Yes	0	1	3	3	1	5
Yes	Yes	0	5	3	3	3	5
Yes	Yes	0	2	4	5	4	3
Yes	Yes	0	3	5	4	3	4
Yes	Yes	0	3	2	5	4	5
Yes	Yes	0	1	4	2	1	4
Yes	Yes	0	5	4	5	5	3
Yes	Yes	0	3	5	5	3	3
Yes	Yes	0	2	3	4	2	3
Yes	Yes	0	3	5	5	2	5
Yes	Yes	0	3	3	3	1	5
Yes	Yes	0	3	5	5	3	3
Yes	Yes	0	1	1	5	1	5
Yes	Yes	0	4	3	4	3	5
Yes	Yes	0	3	3	2	3	2
Yes	Yes	0	4	4	5	4	3
Yes	Yes	0	2	3	2	2	5
Yes	Yes	0	1	5	1	1	5
Yes	Yes	0	3	4	5	3	4
Yes	Yes	0	5	5	5	1	1
Yes	Yes	0	3	4	3	2	5
Yes	Yes	0	4	5	3	3	5
Yes	Yes	0	3	5	5	3	4
		14	.	.	.	.	.
Yes	Yes	0	1	3	2	3	3
Yes	Yes	0	5	5	5	5	5
Yes	Yes	0	4	5	3	5	4
Yes	Yes	0	3	5	5	3	4
		14	.	.	.	.	.
Yes	Yes	0	3	5	3	1	3
Yes	Yes	0	3	3	5	3	4
Yes	Yes	0	2	4	4	2	5
Yes	Yes	0	4	4	5	4	3
Yes	Yes	0	3	3	3	4	3
Yes	Yes	0	1	3	2	1	5
Yes	Yes	0	3	5	3	3	4
Yes	Yes	0	3	1	4	3	5
Yes	Yes	0	4	1	2	2	5
Yes	Yes	0	1	2	3	1	5
Yes	Yes	0	3	5	5	3	5
Yes	Yes	0	4	2	2	4	3
Yes	Yes	0	1	3	2	4	4

Yes	Yes	0	5	4	5	4	4
Yes	Yes	0	2	3	2	2	5
Yes	Yes	0	4	4	4	1	4
Yes	Yes	0	1	3	5	2	4
Yes	Yes	0	4	4	5	4	4
Yes	Yes	0	1	3	5	3	4
Yes	Yes	0	4	3	5	3	5
Yes	Yes	0	3	2	2	2	3
Yes	Yes	0	2	4	4	2	5
Yes	Yes	0	1	1	5	5	1
Yes	Yes	0	1	2	5	2	5
Yes	Yes	0	3	2	2	4	5
Yes	Yes	0	3	4	3	3	4
Yes	Yes	0	3	1	3	1	5
Yes	Yes	0	1	5	1	1	5
Yes	Yes	0	1	5	4	1	5
Yes	Yes	0	3	5	4	3	5
Yes	Yes	0	4	4	4	3	3
Yes	Yes	0	1	3	3	3	3
Yes	Yes	0	3	5	3	5	5
Yes	Yes	0	3	4	4	2	4
Yes	Yes	0	1	4	2	1	5
Yes	Yes	0	1	4	4	3	5
Yes	Yes	0	1	5	5	1	5
Yes	Yes	0	3	4	3	3	5
Yes	Yes	0	1	1	2	1	5
Yes	Yes	0	3	4	5	3	5
Yes	Yes	0	3	4	4	3	4
Yes	Yes	0	5	4	5	5	4
Yes	Yes	0	2	1	3	2	4
Yes	Yes	0	3	4	3	3	4
Yes	Yes	0	5	5	5	5	3
Yes	Yes	0	3	5	5	3	5
Yes	Yes	0	3	3	3	2	4
Yes	Yes	0	1	3	3	1	3
Yes	Yes	0	4	2	4	3	4
Yes	Yes	0	2	4	4	1	3
Yes	Yes	0	3	5	4	3	5
Yes	Yes	0	3	3	3	2	5
Yes	Yes	0	4	5	5	3	4
Yes	Yes	0	2	2	2	2	3
Yes	Yes	0	3	3	5	2	5
Yes	Yes	0	3	5	5	3	4
Yes	Yes	0	1	1	3	1	5
Yes	Yes	0	2	5	2	2	5
Yes	Yes	0	3	3	1	3	2
Yes	Yes	0	3	4	5	3	3
Yes	Yes	0	4	5	5	3	4
Yes	Yes	0	5	5	5	4	3
Yes	Yes	0	3	1	3	2	5

Yes	Yes	0	4	5	5	4	3
Yes	Yes	0	4	5	4	4	4
Yes	Yes	0	3	3	3	3	3
Yes	Yes	0	2	3	2	2	5
Yes	Yes	0	1	2	1	1	5
Yes	Yes	0	4	4	5	3	4
Yes	Yes	0	1	5	3	1	5
Yes	Yes	0	3	4	4	2	5
Yes	Yes	0	3	3	5	2	3
		14	.	.	.	.	.
Yes	Yes	0	4	4	4	4	5
		14	.	.	.	.	.
Yes	Yes	0	3	5	3	3	5
Yes	Yes	1	3	5	5	3	1
Yes	Yes	0	3	1	4	3	2
		14	.	.	.	.	.
Yes	Yes	0	2	3	5	2	5
Yes	Yes	0	3	5	5	1	5
Yes	Yes	0	2	4	4	2	4
Yes	Yes	0	3	3	4	2	5
Yes	Yes	0	1	5	3	2	5
Yes	Yes	0	4	4	3	3	4
Yes	Yes	0	5	2	3	3	5
		14	.	.	.	.	.
Yes	Yes	0	1	5	5	1	5
Yes	Yes	0	2	4	3	2	4
Yes	Yes	0	1	3	4	1	2
Yes	Yes	0	2	3	4	3	2
		14	.	.	.	.	.
Yes	Yes	0	3	1	2	1	4

d7_bbq_q6	d7_bbq_q7	d7_bbq_q8	d7_bbq_q9	d7_bbq_q10	d7_bbq_q11	d7_bbq_q12	d7_bbq_q13
5	1	1	.	5	4	5	5
5	1	1	4	1	1	1	3
5	4	5	4	3	1	3	3
5	1	3	3	3	3	3	3
4	2	1	1	4	3	2	3
5	1	1	1	1	2	3	3
5	4	2	3	3	2	5	3
5	5	2	1	3	3	5	5
5	4	4	3	5	2	4	5
4	2	1	3	4	3	3	2
5	3	1	2	1	3	4	5
4	1	2	3	3	2	3	3
5	1	3	2	3	3	5	4
4	3	4	4	5	5	4	5
4	2	1	3	3	3	5	4
5	2	2	1	3	2	5	5
5	2	1	3	3	2	2	2
3	3	1	3	3	3	3	3
5	1	1	1	5	5	5	5
2	1	1	1	2	1	4	5
4	3	1	2	1	4	3	2
5	1	4	4	3	3	4	4
5	1	2	3	4	1	3	1
5	1	1	3	1	1	5	5
5	3	1	3	4	4	4	5
5	5	1	5	1	5	5	5
5	3	2	3	4	3	3	2
5	1	1	3	4	3	3	4
5	1	3	3	3	2	3	5
.	.	.	.	.	.	.	.
4	2	1	3	2	3	3	3
5	1	1	1	4	1	4	4
3	1	3	3	2	3	3	3
5	3	3	1	4	1	5	4
.	.	.	.	.	.	.	.
5	1	1	3	3	1	5	3
5	2	1	3	3	1	5	3
5	3	1	2	3	2	3	2
5	1	1	1	4	5	4	3
3	3	3	3	3	3	3	3
1	2	1	2	2	1	4	4
4	3	3	3	4	3	4	4
3	1	1	4	4	3	4	4
3	1	1	3	1	4	4	5
5	2	1	3	3	4	4	3
5	3	4	3	3	5	2	4
2	1	1	1	1	3	5	1
2	1	1	2	3	3	4	4

4	2	5	3	5	4	5	4
4	4	3	2	1	3	2	5
4	1	1	3	3	2	4	3
5	1	1	3	4	1	5	5
5	1	1	5	5	2	4	4
4	5	2	3	3	3	3	3
5	1	1	3	1	1	3	3
1	1	2	2	1	1	3	5
5	2	1	1	4	2	2	2
1	1	1	1	1	1	4	1
4	1	1	3	4	1	3	1
3	1	4	3	2	4	2	4
5	2	1	2	3	2	4	4
1	1	1	1	1	3	3	4
5	3	1	1	5	3	5	5
3	1	1	3	5	2	4	2
4	1	1	3	3	4	1	4
4	3	1	1	4	3	4	4
4	3	3	3	2	3	4	3
4	4	1	3	5	4	5	5
4	2	2	4	4	2	4	4
2	1	1	4	2	1	3	2
4	1	1	3	3	4	5	3
5	3	1	5	3	1	5	3
5	3	2	2	3	2	3	4
2	1	1	3	1	1	4	1
5	2	3	3	2	4	4	4
4	5	2	2	4	3	4	4
5	2	1	1	5	2	5	5
5	1	1	3	1	4	1	4
5	5	3	4	4	3	4	4
5	2	4	3	5	4	5	5
5	2	2	3	4	4	3	3
3	2	1	2	1	2	5	5
2	3	1	3	2	3	4	4
4	2	1	3	1	3	3	2
5	1	1	2	1	4	4	3
5	2	2	4	4	3	4	5
2	1	1	2	2	3	3	3
5	2	5	1	5	4	5	5
2	3	3	3	2	2	2	2
5	2	2	2	5	4	5	3
5	1	3	3	4	4	4	5
1	1	1	3	1	1	3	1
5	4	1	3	3	3	4	3
2	3	3	2	3	5	2	3
5	3	2	3	4	2	4	3
5	3	3	4	5	3	5	5
5	2	1	2	5	5	5	3
4	2	2	3	2	3	2	2

5	3	1	1	1	3	3	2
4	3	2	3	4	4	4	3
5	3	1	3	5	3	5	5
5	3	2	4	3	4	4	2
2	3	1	1	1	3	2	2
5	5	3	1	4	4	5	5
4	1	1	4	1	1	5	5
3	1	1	2	3	2	3	4
5	1	1	3	4	3	4	4
.	.	.	.	.	.	.	.
5	1	1	1	3	3	4	5
.	.	.	.	.	.	.	.
5	3	1	3	5	3	5	1
5	.	3	3	3	5	5	5
4	3	3	2	3	4	5	3
.	.	.	.	.	.	.	.
3	1	1	3	2	3	4	3
4	3	1	3	5	2	5	2
5	1	1	1	2	2	4	3
5	3	2	1	5	3	4	4
3	2	3	4	4	2	5	5
5	1	1	4	4	3	4	4
4	1	1	3	3	2	5	3
.	.	.	.	.	.	.	.
5	1	3	3	5	1	5	5
5	1	1	3	5	3	3	3
2	1	1	3	1	1	2	4
4	2	1	4	3	2	2	2
.	.	.	.	.	.	.	.
3	1	1	4	1	3	1	2

d7_bbq_q14	d7_bbq	d7_fabq_phys	d7_fabq_work	d7_fabq	d7_quebec	d7_tampa	d7_had_dep
5	13	21	31	52	81	63	13
3	34	8	27	35	82	34	3
5	16	22	42	64	28	52	7
4	22	15	34	49	71	51	10
4	24	18	37	55	51	48	4
4	26	17	14	31	47	47	9
4	25	5	24	29	54	39	12
5	18	16	13	29	71	54	12
5	15	16	.	.	48	57	10
4	24	4	35	39	34	41	10
5	23	19	38	57	41	53	6
3	29	15	31	46	23	38	6
4	16	22	34	56	30	45	7
5	14	12	37	49	15	48	4
5	23	14	22	36	40	46	7
5	16	17	42	59	67	.	8
3	29	8	14	22	46	35	5
3	25	24	42	66	37	51	4
5	21	17	36	53	46	.	13
4	25	8	41	49	62	37	12
5	30	18	24	42	63	.	10
5	16	19	8	27	62	48	11
4	28	10	26	36	53	44	14
5	25	24	39	63	33	50	.
5	18	20	7	27	34	46	6
5	17	18	42	60	37	52	9
4	24	11	16	27	25	45	3
5	20	17	17	34	34	50	8
5	17	17	33	50	56	48	9
.	.	.	.	.	.	.	.
3	32	12	0	12	37	38	5
3	18	7	32	39	15	27	6
3	25	12	14	26	19	32	3
5	15	15	16	31	44	49	18
.	.	.	.	.	.	.	.
5	21	21	30	51	69	46	11
5	21	18	35	53	46	43	9
4	26	14	9	23	42	43	9
5	19	18	34	52	36	50	3
3	27	17	42	59	33	42	12
3	33	12	39	51	26	37	3
4	20	17	24	41	37	46	11
3	27	11	9	20	12	41	5
1	32	4	0	4	21.0526	30	5
3	29	13	13	26	3	45	5
5	18	13	25	38	45	37	8
3	33	13	24	37	31	.	5
3	31	13	41	54	18	33	3

4	13	21	40	61	41	60	5
4	28	3	4	7	7	34	2
4	23	19	30	49	72	40	6
5	20	20	10	30	19	51	2
4	18	20	42	62	42	49	12
3	27	16	9	25	42	48	4
3	26	15	11	26	25	36	7
4	31	10	12	22	7.3684	46	8
5	25	7	40	47	50	40	8
1	38	12	8	20	25	37	4
4	29	6	20	26	21	36	10
3	29	13	21	34	12	43	.
3	24	12	28	40	27	41	10
3	34	2	6	8	36	32	5
3	23	19	39	58	55	52	12
4	25	3	32	35	28	37	6
2	27	17	29	46	32	38	8
4	21	14	36	50	34	46	7
4	27	13	14	27	24	44	5
5	18	14	38	52	36	46	11
4	21	19	29	48	27	46	7
3	34	12	6	18	19	39	2
5	24	16	33	49	39	42	3
5	21	20	15	35	12.6316	53	5
5	22	12	9	21	16	49	11
2	39	5	6	11	21	23	2
4	20	14	20	34	20	43	2
4	21	18	33	51	20	45	9
5	14	19	7	26	45	52	9
3	33	16	7	23	21	39	7
4	20	14	39	53	54	52	12
5	10	24	42	66	55	66	9
5	19	21	21	42	54	45	11
2	28	9	30	39	56	44	7
3	31	16	27	43	15	41	2
3	30	4	8	12	22	36	4
3	27	17	17	34	55	52	10
5	17	19	34	53	21	40	7
3	31	14	10	24	29	26	6
5	10	24	38	62	53	52	10
2	35	7	7	14	62	37	15
3	20	19	42	61	33	48	14
5	15	12	36	48	22	49	11
1	41	12	2	14	24	21	2
1	28	9	17	26	59	35	12
1	33	18	28	46	25	48	8
4	20	17	25	42	51	46	4
5	12	16	30	46	53	52	12
3	17	15	39	54	72	51	8
3	32	20	14	34	28	37	1

4	24	20	26	46	31	45	7
4	20	18	12	30	22	39	4
5	19	18	21	39	54	51	6
5	26	5	21	26	22	45	6
2	40	7	20	27	6	31	3
5	14	19	40	59	63	51	7
4	25	17	38	55	24	40	2
4	25	8	11	19	7	37	8
4	21	22	30	52	37	49	9
.	.	.	.	.	.	.	.
5	19	8	11	19	63	38	11
.	.	.	.	.	.	.	.
3	23	20	30	50	34	47	5
5	15	20	34	54	73.6842	.	13
3	25	18	42	60	41	48	10
.	.	.	.	.	.	.	.
2	29	10	31	41	44	33	4
2	22	12	17	29	29	52	2
4	25	19	27	46	.	50	6
4	20	11	22	33	14	51	4
5	20	11	15	26	43	46	12
3	22	18	31	49	51	37	8
4	24	7	11	18	22	38	8
.	.	.	.	.	.	.	.
5	15	24	36	60	46	46	10
3	25	5	3	8	82	32	10
3	33	0	36	36	66	45	2
3	30	15	30	45	34	41	4
.	.	.	.	.	.	.	.
1	39	9	3	12	28	32	2

d7_had_anx	d7_dallas_daily	d7_dallas_work	d7_dallas_anxdep	d7_dallas_social	d30_attend	d30_bbq_coll
17	82.2	74	74	72	Yes	Yes
5	67.2	74	20	38	No	
9	60.6	67	60	67	Yes	Yes
11	73.2	81	60	55	Yes	Yes
9	69.6	61	46	34	Yes	Yes
14	73.2	67	60	60	Yes	Yes
13	46.2	74	47	28	No	
13	69.6	60	60	31	Yes	Yes
13	61.2	.	60	60	Yes	Yes
13	52.2	74	60	54	Yes	Yes
10	49.8	60	40	40	Yes	Yes
10	55.2	48	40	14	Yes	Yes
6	46.2	41	53	33	Yes	Yes
7	43.8	33	7	13	Yes	Yes
4	45	20	0	12	Yes	Yes
9	61.8	74	46	47	Yes	Yes
11	64.8	66	60	67	No	
4	58.8	61	6	25	Yes	Yes
15	64.8	81	80	74	Yes	Yes
15	64.2	67	74	46	Yes	Yes
15	70.2	60	60	60	No	
15	73.2	60	73	73	No	
21	78	94	86	80	Yes	Yes
.	49.2	74	60	80	Yes	Yes
10	50.4	53	47	40	Yes	Yes
11	51	34	19	14	Yes	Yes
11	46.8	33	40	47	Yes	Yes
17	43.8	80	67	61	Yes	Yes
7	72.6	67	60	59	Yes	Yes
.	.	.	.	.	No	
8	49.2	39	40	33	Yes	Yes
15	39	14	68	0	Yes	Yes
3	36	25	0	0	Yes	Yes
6	58.2	60	60	80	Yes	Yes
.	.	.	.	.	No	
12	63.6	81	53	60	Yes	Yes
8	62.4	74	60	53	Yes	Yes
10	69.6	47	40	33	Yes	Yes
9	67.2	53	20	32	Yes	Yes
12	52.2	60	60	60	No	
7	26.4	34	25	20	Yes	No
7	57.6	61	60	61	Yes	Yes
5	37.8	20	14	7	Yes	Yes
4	44.4	14	13	7	Yes	Yes
8	32.4	27	26	0	Yes	Yes
20	58.2	67	68	47	Yes	Yes
5	55.2	60	33	0	Yes	Yes
14	38.4	46	21	12	No	

16	53.4	67	100	34	Yes	Yes
4	38.4	46	6	14	Yes	Yes
5	72.6	53	0	12	Yes	Yes
6	43.8	34	20	13	Yes	Yes
11	61.8	68	67	26	Yes	Yes
11	56.4	48	13	19	Yes	Yes
5	42.6	67	27	13	Yes	Yes
5	40.2	27	33	27	Yes	Yes
11	49.2	74	66	73	Yes	Yes
6	49.8	47	20	12	Yes	Yes
8	52.2	35	46	35	Yes	Yes
.	82.2	87	32	33	Yes	Yes
9	37.8	61	20	13	Yes	Yes
11	52.2	60	46	19	Yes	Yes
13	61.2	61	54	60	Yes	Yes
9	53.4	80	6	40	Yes	Yes
8	68.4	61	26	19	Yes	Yes
10	64.2	67	53	20	Yes	Yes
6	55.2	47	27	12	Yes	Yes
16	64.2	67	67	53	Yes	Yes
11	58.2	60	33	33	Yes	Yes
11	44.4	39	32	6	Yes	Yes
4	39	54	6	12	Yes	Yes
10	49.8	40	26	37	Yes	Yes
9	52.2	40	41	40	Yes	Yes
4	30	7	0	6	Yes	Yes
10	41.4	26	14	13	Yes	Yes
9	55.8	54	26	34	Yes	Yes
11	70.2	53	60	59	Yes	Yes
7	47.4	46	34	34	Yes	Yes
14	75.6	74	60	54	Yes	Yes
10	84.6	68	94	87	Yes	Yes
16	76.2	53	54	46	Yes	Yes
13	43.2	47	26	21	Yes	Yes
6	36	33	6	0	Yes	Yes
5	47.4	33	34	21	Yes	Yes
13	67.2	60	53	53	Yes	Yes
11	41.4	34	33	0	Yes	Yes
7	55.2	46	47	6	Yes	Yes
18	52.8	53	73	40	Yes	Yes
15	76.2	87	80	67	Yes	Yes
9	58.2	61	53	39	Yes	Yes
10	61.8	55	40	32	Yes	Yes
12	49.2	32	0	12	Yes	Yes
8	64.2	60	53	46	Yes	Yes
9	25.8	40	26	14	Yes	Yes
5	70.2	60	25	13	Yes	Yes
10	47.4	60	87	60	Yes	Yes
7	78.6	87	39	67	Yes	Yes
8	49.2	27	13	21	Yes	Yes

7	49.2	47	20	20	Yes	Yes
10	43.8	40	33	6	Yes	Yes
10	73.8	54	33	72	Yes	Yes
13	49.2	54	40	60	Yes	Yes
9	36	35	7	12	Yes	Yes
15	78.6	74	67	20	Yes	Yes
3	43.8	34	13	0	Yes	Yes
15	22.2	39	34	21	Yes	Yes
7	64.2	54	60	40	No	
.	.	.	.	.	No	
10	68.4	54	33	54	No	
.	.	.	.	.	No	
11	58.8	73	60	67	No	
13	67.2	53	54	47	No	
12	61.8	67	46	39	No	
.	.	.	.	.	No	
9	67.2	67	33	47	Yes	Yes
7	37.8	39	12	6	Yes	Yes
12	73.2	74	80	87	Yes	Yes
7	48.6	47	26	20	No	
16	58.2	67	67	61	No	
11	61.2	60	33	47	No	
8	46.2	47	20	40	No	
.	.	.	.	.	No	
16	62.4	53	67	33	Yes	Yes
10	61.2	60	46	54	No	
7	79.2	80	66	26	No	
14	49.8	53	7	0	No	
.	.	.	.	.	No	
9	52.8	13	0	6	No	

d30_bbq_comp	d30_bbq_nfill	d30_bbq	d30_fabq_phys	d30_fabq_work	d30_fabq	d30_quebec
Yes	0	20	17	.	.	53
	14	.	.	.	.	.
Yes	0	32	2	.	.	23
Yes	0	20	16	38	54	.
Yes	0	27	9	42	51	44.2105
Yes	0	41	2	11	13	12
	14	.	.	.	.	.
Yes	0	19	2	14	16	37.8947
Yes	0	18	10	.	.	52
Yes	0	21	16	39	55	31
Yes	0	28	8	38	46	52
Yes	0	27	13	30	43	24
Yes	0	19	21	.	.	17
Yes	0	26	5	36	41	5
Yes	0	22	13	34	47	26
Yes	0	18	10	42	52	30
	14	.	.	.	.	.
Yes	0	28	18	42	60	28
Yes	0	26	1	40	41	23
Yes	0	23	20	34	54	45
	14	.	.	.	.	.
	14	.	.	.	.	.
Yes	0	28	12	18	30	42
Yes	0	41	0	25	25	3
Yes	0	25	15	0	15	26
Yes	0	19	2	40	42	22
Yes	0	25	6	8	14	12
Yes	0	21	1	4	5	14
Yes	0	29	2	19	21	22
	14	.	.	.	.	.
Yes	0	30	10	0	10	19
Yes	0	25	7	37	44	12
Yes	0	22	4	18	22	5
Yes	0	37	2	2	4	18
	14	.	.	.	.	.
Yes	0	23	22	36	58	72
Yes	0	21	13	38	51	48
Yes	0	25	12	10	22	36
Yes	0	11	24	42	66	74
	14	.	.	.	.	.
No	14	.	.	.	.	.
Yes	0	31	10	18	28	11
Yes	0	23	0	8	8	2
Yes	1	36	2	0	2	14
Yes	0	30	13	2	15	2
Yes	0	21	15	24	39	27
Yes	0	37	2	12	14	17
	14	.	.	.	.	.

Yes	0	30	12	22	34	22
Yes	0	33	0	4	4	3
Yes	0	30	11	29	40	54
Yes	0	30	12	10	22	13
Yes	0	21	16	42	58	38
Yes	0	34	0	0	0	15
Yes	1	27	16	20	36	13
Yes	0	44	0	4	4	2.1053
Yes	0	24	3	21	24	27
Yes	0	32	8	9	17	20
Yes	0	30	5	17	22	4
Yes	0	20	10	4	14	11
Yes	0	30	4	15	19	17
Yes	0	35	12	15	27	18
Yes	0	25	12	29	41	52
Yes	0	27	2	38	40	25
Yes	0	29	12	23	35	24
Yes	0	24	5	32	37	34
Yes	0	30	3	8	11	15
Yes	0	29	3	25	28	24
Yes	0	23	6	28	34	19
Yes	0	35	6	4	10	14
Yes	0	40	5	29	34	13
Yes	0	33	1	18	19	7.3684
Yes	0	23	7	11	18	2
Yes	0	43	0	2	2	20
Yes	0	27	16	12	28	18
Yes	0	20	16	35	51	25
Yes	0	22	15	7	22	50
Yes	0	32	2	5	7	14
Yes	0	28	5	42	47	38
Yes	0	11	21	42	63	54
Yes	0	25	12	15	27	35
Yes	0	43	0	8	8	11
Yes	0	37	5	18	23	3
Yes	0	33	1	3	4	14
Yes	0	37	0	5	5	7
Yes	0	26	15	27	42	18
Yes	0	31	9	3	12	27
Yes	0	19	0	40	40	51
Yes	0	33	0	36	36	53
Yes	0	29	2	42	44	23
Yes	0	41	0	42	42	0
Yes	0	41	8	0	8	21
Yes	0	29	8	17	25	54
Yes	0	23	6	21	27	8
Yes	0	22	16	28	44	43
Yes	0	20	11	31	42	49
Yes	0	20	6	33	39	53
Yes	0	39	8	2	10	1

Yes	0	26	0	15	15	15.7895
Yes	0	44	7	2	9	4
Yes	0	25	13	17	30	49
Yes	0	32	0	19	19	16
Yes	0	38	3	13	16	8
Yes	0	18	15	41	56	41
Yes	0	25	2	28	30	9
Yes	0	30	4	31	35	0
	14	.	.	.	.	.
	14	.	.	.	.	.
	14	.	.	.	.	.
	14	.	.	.	.	.
	14	.	.	.	.	.
	14	.	.	.	.	.
	14	.	.	.	.	.
	14	.	.	.	.	.
Yes	0	35	5	29	34	35
Yes	0	37	1	10	11	9
Yes	0	25	17	23	40	40
	14	.	.	.	.	.
	14	.	.	.	.	.
	14	.	.	.	.	.
	14	.	.	.	.	.
	14	.	.	.	.	.
Yes	0	21	24	33	57	50.5263
	14	.	.	.	.	.
	14	.	.	.	.	.
	14	.	.	.	.	.
	14	.	.	.	.	.
	14	.	.	.	.	.

d30_tampa	d1d7_bbq	d1d7_fabq	d1d7_tampa	d7d30_bbq	d7d30_fabq_phys	d7d30_fabq_work
44	1	20	16	7	-4	.
.	7	-6	-1	.	.	.
35	0	7	1	16	-20	.
.	4	-10	6	-2	1	4
.	5	-4	-7	3	-9	5
21	5	-12	-4	15	-15	-3
.	4	-12	-4	.	.	.
41	-5	.	.	1	-14	1
50	-1	.	4	3	-6	.
43	11	-20	-4	-3	12	4
40	3	6	1	5	-11	0
46	-7	7	2	-2	-2	-1
42	-2	9	0	3	-1	.
32	-4	-6	-5	12	-7	-1
41	2	-3	5	-1	-1	12
38	2	-2	.	2	-7	0
.	7	2	2	.	.	.
44	.	.	.	3	-6	0
39	2	-3	.	5	-16	4
40	0	-14	4	-2	12	-7
.	.	9	.	.	.	.
.	-4	-20	.	.	.	.
39	4	-3	1	0	2	-8
30	-12	.	-6	16	-24	-14
44	2	7	-6	7	-5	-7
39	0	14	3	2	-16	-2
40	7	8	-8	1	-5	-8
.	3	20	-2	1	-16	-13
37	2	-7	-6	12	-15	-14
.	.	.	.	.	.	.
33	0	-8	-3	-2	-2	0
33	3	1	-6	7	0	5
25	1	-1	-6	-3	-8	4
31	1	-13	-3	22	-13	-14
.	.	.	.	.	.	.
49	1	0	4	2	1	6
41	2	-2	6	0	-5	3
42	-3	1	2	-1	-2	1
56	1	-1	11	-8	6	8
.	3	11	-2	.	.	.
.	4	7	5	.	.	.
33	0	0	-1	11	-7	-6
29	7	3	4	-4	-11	-1
28	1	-3	-13	4	-2	0
37	-2	-2	7	1	0	-11
26	-4	-1	4	3	2	-1
34	13	-21	.	4	-11	-12
.	2	0	-5	.	.	.

39	2	4	7	17	-9	-18
23	5	-19	4	5	-3	0
36	-5	3	3	7	-8	-1
38	-1	5	-4	10	-8	0
49	2	-1	-5	3	-4	0
24	-1	4	-2	7	-16	-9
33	2	-23	1	1	1	9
29	6	-7	12	13	-10	-8
23	7	-13	-15	-1	-4	-19
36	1	-23	-3	-6	-4	1
28	6	-14	-3	1	-1	-3
43	4	9	-2	-9	-3	-17
33	-6	-6	6	6	-8	-13
31	6	.	.	1	10	9
45	2	1	-7	2	-7	-10
32	1	-11	-2	2	-1	6
36	4	-8	-1	2	-5	-6
43	3	-15	-6	3	-9	-4
36	-3	0	-1	3	-10	-6
45	-4	28	1	11	-11	-13
47	8	0	-6	2	-13	-1
30	0	-2	-1	1	-6	-2
32	1	5	-2	16	-11	-4
29	-4	-16	7	12	-19	3
35	1	-6	.	1	-5	2
19	3	7	-2	4	-5	-4
33	-4	4	0	7	2	-8
43	1	3	3	-1	-2	2
42	-5	0	5	8	-4	0
34	-1	1	-2	-1	-14	-2
44	2	-11	3	8	-9	3
63	-2	1	6	1	-3	0
32	-2	2	1	6	-9	-6
34	0	4	4	15	-9	-22
31	2	2	1	6	-11	-9
.	4	-9	4	3	-3	-5
26	4	8	2	10	-17	-12
40	0	-1	-9	9	-4	-7
29	10	-2	-9	0	-5	-7
49	-10	8	6	9	-24	2
44	22	-44	-14	-2	-7	29
36	0	-2	2	9	-17	0
23	-4	-3	2	26	-12	6
22	10	-4	1	0	-4	-2
32	-3	-12	4	1	-1	0
26	10	2	10	-10	-12	-7
45	-1	-2	4	2	-1	3
45	-7	.	1	8	-5	1
41	-3	8	7	3	-9	-6
24	7	-3	-7	7	-12	-12

39	-1	10	2	2	-20	-11
24	-2	.	10	24	-11	-10
48	-10	7	3	6	-5	-4
47	-5	-17	-3	6	-5	-2
30	9	-3	3	-2	-4	-7
46	0	-1	-2	4	-4	1
35	-1	18	-3	0	-15	-10
28	7	-32	.	5	-4	20
.	3	8	6	.	.	.
.	.	.	.	.	.	.
.	-3	-10	-1	.	.	.
.	.	.	.	.	.	.
.	5	-3	-1	.	.	.
.	1	1	.	.	.	.
.	4	9	1	.	.	.
.	.	.	.	.	.	.
29	3	-8	-11	6	-5	-2
36	-13	29	22	15	-11	-7
36	-5	0	4	0	-2	-4
.	-2	-8	6	.	.	.
.	2	-22	3	.	.	.
.	1	-7	-9	.	.	.
.	-9	6	1	.	.	.
.	.	.	.	.	.	.
.	-2	0	.	6	0	-3
.	-4	-9	1	.	.	.
.	15	-30	-6	.	.	.
.	15	-15	-11	.	.	.
.	.	.	.	.	.	.
.	4	0	-4	.	.	.

d7d30_fabq	d7d30_tampa	d7d30_quebec
.	-19	-28
.	.	.
.	-17	-5
5	.	.
-4	.	-6.7895
-18	-26	-35
.	.	.
-13	-13	-33.1053
.	-7	4
16	2	-3
-11	-13	11
-3	8	1
.	-3	-13
-8	-16	-10
11	-5	-14
-7	.	-37
.	.	.
-6	-7	-9
-12	.	-23
5	3	-17
.	.	.
.	.	.
-6	-5	-11
-38	-20	-30
-12	-2	-8
-18	-13	-15
-13	-5	-13
-29	.	-20
-29	-11	-34
.	.	.
-2	-5	-18
5	6	-3
-4	-7	-14
-27	-18	-26
.	.	.
7	3	3
-2	-2	2
-1	-1	-6
14	6	38
.	.	.
.	.	.
-13	-13	-26
-12	-12	-10
-2	-2	-7.0526
-11	-8	-1
1	-11	-18
-23	.	-14
.	.	.

-27	-21	-19
-3	-11	-4
-9	-4	-18
-8	-13	-6
-4	0	-4
-25	-24	-27
10	-3	-12
-18	-17	-5.2632
-23	-17	-23
-3	-1	-5
-4	-8	-17
-20	0	-1
-21	-8	-10
19	-1	-18
-17	-7	-3
5	-5	-3
-11	-2	-8
-13	-3	0
-16	-8	-9
-24	-1	-12
-14	1	-8
-8	-9	-5
-15	-10	-26
-16	-24	-5.2632
-3	-14	-14
-9	-4	-1
-6	-10	-2
0	-2	5
-4	-10	5
-16	-5	-7
-6	-8	-16
-3	-3	-1
-15	-13	-19
-31	-10	-45
-20	-10	-12
-8	.	-8
-29	-26	-48
-11	0	-3
-12	3	-2
-22	-3	-2
22	7	-9
-17	-12	-10
-6	-26	-22
-6	1	-3
-1	-3	-5
-19	-22	-17
2	-1	-8
-4	-7	-4
-15	-10	-19
-24	-13	-27

-31	-6	-15.2105
-21	-15	-18
-9	-3	-5
-7	2	-6
-11	-1	2
-3	-5	-22
-25	-5	-15
16	-9	-7
.	.	.
.	.	.
.	.	.
.	.	.
.	.	.
.	.	.
.	.	.
.	.	.
-7	-4	-9
-18	-16	-20
-6	-14	.
.	.	.
.	.	.
.	.	.
.	.	.
.	.	.
-3	.	4.5263
.	.	.
.	.	.
.	.	.
.	.	.
.	.	.
